# Mindfulness-Based Interventions for Bereavement: A Systematized Narrative Review

**DOI:** 10.3390/healthcare14050673

**Published:** 2026-03-06

**Authors:** Fabio D’Antoni, Fabio Mattiussi, Cristiano Crescentini

**Affiliations:** 1 Child and Family Service, Azienda Sanitaria Universitaria Friuli Centrale (ASUFC), 33100 Udine, Italy; 2 School of Psychology, University of Padova, 35131 Padova, Italy; 3 Department of Languages and Literatures, Communication, Education and Society, University of Udine, 33100 Udine, Italy; cristiano.crescentini@uniud.it

**Keywords:** bereavement, grief, mindfulness-based interventions, prolonged grief disorder, mental health

## Abstract

Background/Objectives: Mindfulness-based interventions (MBIs) have shown promising effects across diverse areas of psychiatry, yet their specific role in bereavement remains insufficiently synthesized. Bereavement is a universal but heterogeneous process, with a minority of individuals at risk of developing prolonged grief disorder (PGD). Understanding the potential benefits of MBIs in this context is crucial for informing clinical practice. Methods: Following the methodological framework of systematized narrative reviews, a comprehensive literature search was conducted across major databases. Eligible studies included empirical investigations of MBIs applied to bereavement, without restrictions on type of loss, population, or intervention format. Data were narratively synthesized and summarized in tables; no meta-analysis was performed. Results: Seventeen studies met inclusion criteria. The strongest evidence was found for Mindfulness-Based Stress Reduction (MBSR) and Mindfulness-Based Cognitive Therapy (MBCT), which demonstrated improvements in grief-related distress, depressive symptoms, and psychological well-being. Across interventions, MBIs were associated with reductions in rumination and experiential avoidance, increased self-compassion, and enhanced emotion regulation. However, most studies were limited by small sample sizes, heterogeneous outcome measures, and a lack of long-term follow-up. Conclusions: MBIs show promise as adjunctive interventions in bereavement care, targeting mechanisms central to grief adaptation, including acceptance, decentering, and self-compassion. Nevertheless, the evidence remains preliminary and methodologically constrained. Future high-quality randomized controlled trials are needed to establish efficacy, clarify mechanisms of action, and define the role of MBIs alongside established grief therapies.

## 1. Introduction

### 1.1. Mindfulness-Based Interventions

Mindfulness-Based Interventions (MBIs) refer to a broad spectrum of structured psychological programs and meditation practices unified by the cultivation of mindful-ness—commonly defined as “the awareness that emerges through paying attention, on purpose, in the present moment, and nonjudgmentally” [1]. This operationalization has been further refined into two interrelated components: the self-regulation of attention toward present-moment experience and an orientation of openness and acceptance [2].

Among the most established protocols are Mindfulness-Based Stress Reduction (MBSR) [3] and Mindfulness-Based Cognitive Therapy (MBCT) [4,5], typically delivered as secular, manualized group programs of eight weekly sessions plus a retreat [6]. These interventions integrate formal practices (e.g., sitting meditation, body scan, mindful movement) with informal practices designed to bring awareness into daily life [7]. The term MBI has also been extended to include “third wave” approaches such as Dialectical Behavior Therapy (DBT) [8] and Acceptance and Commitment Therapy (ACT) [9], which incorporate mindfulness skills even when not centered on formal meditation [10].

MBIs are extensively utilized in the psychiatric domain for treating a broad spectrum of psychological and psychiatric problems [10]. Across various patient populations, MBIs generally produce small-to-large reductions (standardized mean difference = 0.21–0.89) in common psychological symptoms, including depression, anxiety, and stress, relative to no treatment controls [11]. MBCT was developed specifically for psychiatric application and has demonstrated consistent efficacy for preventing relapse in major depressive disorder. MBSR and other MBIs show broader utility in reducing current anxiety and mood symptoms in patients with affective disorders [7].

MBIs are unified by the cultivation of mindfulness as a core mechanism of change across diverse clinical applications [6,10]. Their clinical utility can be understood in terms of both specific psychological mechanisms and common therapeutic factors. At the psychological level, MBIs foster (1) acceptance and non-reactivity, encouraging an open and nonjudgmental stance toward inner experience [7]; (2) meta-awareness and decentering, the capacity to observe thoughts and emotions as transient mental events rather than facts, thereby reducing identification with maladaptive patterns; (3) enhanced emotion regulation and cognitive flexibility, which diminish rumination and worry; and (4) positive psychological resources, such as self-compassion and positive affect, linked to greater well-being [6,7]. Beyond these mechanisms, MBIs also benefit from common therapeutic factors: the therapeutic alliance, strengthened when instructors embody mindfulness qualities; the group context, which provides social support and shared ritual; and the treatment rationale and expectancy, which frame mindfulness practice as an active pathway to relief and resilience [11]. Together, these ingredients explain the transdiagnostic effectiveness of MBIs in fostering adaptation and alleviating distress.

### 1.2. Bereavement

Bereavement refers to the universal yet highly individualized experience that follows the death of a significant other. Psychologically, it represents a multidimensional response activated by the disruption of a meaningful bond, encompassing affective, cognitive, behavioral, relational, and existential components. Although bereavement is a normative human experience, its expression and trajectory are shaped by personal, relational, cultural, and situational factors. The earliest psychological conceptualization of bereavement emerged within psychoanalysis. Freud (1917), in Mourning and Melancholia, described mourning as a process of gradual withdrawal of libidinal investment from the lost object [12]. A major paradigm shift occurred with attachment theory. Bowlby (1980) conceptualized grief as a biologically grounded response to separation from an attachment figure [13]. Parkes (1972) and Bowlby (1980) described sequential phases including shock, yearning and searching, disorganization, and reorganization [13,14]. While stage-based models such as Kübler-Ross’s five stages (1969) gained widespread influence, they have been criticized for oversimplifying and rigidifying a fundamentally non-linear process [15,16].

Later models reframed mourning as an active adaptive process. Worden (2002) proposed four tasks of mourning, emphasizing acceptance of the loss, emotional processing, adaptation to an altered world, and redefinition of the ongoing relationship [17]. Rubin’s Two-Track Model distinguished between biopsychosocial functioning and the continuing inner relationship with the deceased [18,19]. The Dual Process Model, grounded in stress and coping theory, conceptualized mourning as oscillation between loss-oriented and restoration-oriented coping [20]. Constructivist approaches further emphasized meaning-making processes. Neimeyer (2001) described bereavement as a reconstruction of disrupted meaning systems, involving sense-making, benefit-finding, and identity reorganization [21]. Similarly, the Continuing Bonds framework demonstrated that maintaining an adaptive internalized relationship with the deceased is not pathological but can facilitate integration [22].

Although bereavement is a universal human experience, its trajectory varies considerably; most individuals adapt over time, yet a significant minority experience persistent and debilitating symptoms consistent with Prolonged Grief Disorder (PGD) [23]. PGD, previously referred to as “complicated grief” or “persistent complex bereavement disorder,” is now formally recognized in both the ICD-11 and the DSM-5-TR [24]. It is characterized by intense yearning and longing for the deceased, persistent preoccupation with thoughts or memories of the lost person, and clinically significant functional impairment. Additional associated features include identity disruption (e.g., feeling that part of oneself has died), emotional numbness, intense emotional pain such as bitterness or anger, and difficulty reintegrating into social, occupational, or relational roles [25]. These symptoms exceed expected cultural or religious norms and result in marked impairment in daily functioning.

PGD is typically assessed using validated self-report and clinician-administered instruments. Commonly used measures include the Prolonged Grief Disorder-13 (PG-13), a 13-item scale aligned with ICD-11 criteria; the Inventory of Complicated Grief (ICG), a 19-item measure of symptom severity (with scores ≥ 25–30 indicating clinically significant impairment); the Brief Grief Questionnaire (BGQ), a 5-item screening tool; and the Structured Clinical Interview for Complicated Grief (SCI-CG), a clinician-administered diagnostic interview [25].

Prevalence estimates suggest that PGD affects approximately 9.8% to 34.3% of be-reaved individuals, depending on population and context [24]. Certain forms of loss, such as perinatal bereavement, are associated with substantially elevated risk, with rates nearly three times higher than other bereavement types [26]. PGD is also associated with significant adverse outcomes, including increased risk of suicidal ideation and behavior, as well as physical health conditions such as cardiovascular disease, cancer, hypertension, and immune dysregulation [25].

Given its prevalence, functional burden, and health implications, PGD represents a major clinical concern and underscores the need for interventions targeting core maintaining mechanisms such as rumination, experiential avoidance, emotional dysregulation, and self-criticism—processes that mindfulness-based approaches may specifically address.

### 1.3. Mindfulness and Bereavement

MBIs may support both normative and pathological grief by targeting mechanisms central to adaptation [26]: enhancing present-moment awareness, reducing rumination and avoidance [27,28], and fostering acceptance [29] and continuing bonds with the de-ceased [30]. These processes can mitigate maladaptive cognitive-emotional cycles and promote flexibility in the confrontation–avoidance oscillation typical of grief [19].

At the psychological level, mindfulness may counteract experiential avoidance by cultivating non-judgmental present-moment awareness, enabling bereaved individuals to remain in contact with painful emotions without compulsive suppression or withdrawal [31]. This stance may be particularly relevant in PGD, where persistent avoidance of grief-related affect paradoxically maintains distress. Mindfulness also helps to interrupt rumination by strengthening attentional flexibility and fostering decentering—the capacity to observe thoughts as transient mental events rather than as definitive truths about the self or the loss [32]. By weakening repetitive self-referential loops, MBIs may reduce the cognitive entrenchment characteristic of prolonged grief. In addition, mindfulness-based approaches cultivate self-compassion, which may mitigate guilt and shame frequently observed in traumatic forms of bereavement. Through self-kindness and recognition of common humanity, individuals may soften harsh self-criticism and reduce over-identification with narratives of failure or responsibility [31].

### 1.4. Aims of the Study

Despite encouraging findings in various psychiatric contexts, the evidence base for MBIs in bereavement remains fragmented, heterogeneous, and methodologically limited. Indeed, while several studies report improvements in grief-related distress and associated psychological outcomes following MBIs, the evidence remains methodologically diverse, with variability in study design, populations, intervention formats, and outcome measures. Notably, some comparative trials suggest that grief-focused cognitive-behavioral therapies may yield stronger effects on primary grief outcomes, whereas mindfulness-based approaches appear to exert more consistent benefits on transdiagnostic processes such as rumination, emotional regulation, and self-compassion. These mixed findings underscore the need for a structured synthesis of the available literature.

Therefore, the purpose of this article is to provide a systematized narrative review of MBIs in bereavement, in light of the growing clinical interest in mindfulness as an adjunct or alternative to established grief therapies. Specifically, this review seeks to examine (1) the current evidence regarding the feasibility, safety, and potential efficacy of MBIs in be-reaved populations; (2) the extent to which grief-specific versus transdiagnostic outcomes are affected; and (3) the methodological strengths and limitations that characterize the existing body of research. Given the heterogeneity of the literature, the review adopts an exploratory rather than hypothesis-testing approach.

## 2. Materials and Methods

### 2.1. Search Strategy and Selection Criteria

This review followed the methodological framework of systematized narrative reviews [33,34], which combine elements of systematic reviews (explicit search strategy, eligibility criteria, PRISMA flow diagram) with the flexibility of narrative synthesis. Electronic searches were performed in PubMed/MEDLINE, PsycINFO, Scopus, and Web of Science Core Collection from 1 January 2000, to 9 August 2025. Search strings combined mindfulness terms (“mindfulness,” “mindfulness-based,” “mindfulness meditation,” “mindfulness-based stress reduction/MBSR,” “mindfulness-based cognitive therapy/MBCT,” “mindful self-compassion/MSC,” “acceptance and commitment therapy/ACT”) with bereavement terms (“grief,” “bereavement,” “mourning,” “complicated grief,” “prolonged grief,” “prolonged grief disorder”), using database-specific field codes and controlled vocabulary where available. Searches were limited to peer-reviewed articles in English involving human participants.

### 2.2. Eligibility Criteria

Inclusion and exclusion criteria were defined a priori:

- Population: Adults bereaved by the death of a loved one. Eligible populations included perinatal loss (miscarriage, stillbirth), suicide, homicide, spousal/partner, child, parent, or heterogeneous bereavement samples.
  ○ Included: bereavement due to death, across different causes.
  ○ Excluded: losses unrelated to death (e.g., chronic pain, job loss, identity loss); “professional grief” in healthcare providers when not related to personal bereavement.
  ○ Ambiguous loss (e.g., disappearance of a relative) was excluded for conceptual consistency with the definition of bereavement as loss through death.
- Intervention: Structured MBIs, including manualized protocols such as MBSR, MBCT, and retreats with explicit mindfulness training. ACT-based programs were included only when mindfulness constituted a central therapeutic component, rather than a superficial mention.
  ○ Excluded: interventions mentioning mindfulness superficially without structured practice or protocol.
- Study design: Empirical studies, including randomized controlled trials (RCTs), non-randomized controlled trials, pre–post cohort studies, and qualitative investigations exploring participant experiences.
  ○ Excluded: study protocols, editorials, conference abstracts, scoping or narrative reviews (these were retained for background only).
- Outcomes: Studies were eligible if they assessed either grief-specific outcomes (e.g., Prolonged Grief Disorder, Inventory of Complicated Grief, Texas Revised Inventory of Grief) or clinically relevant secondary outcomes (e.g., depression, anxiety, PTSD symptoms, emotion regulation, mindfulness, self-compassion, quality of life). Studies lacking a grief-specific outcome were included but explicitly annotated and downgraded in quality appraisal.
- Language: Only articles published in English were included.

When multiple publications referred to the same participant cohort, these were all retained if they reported complementary findings (e.g., quantitative outcomes and qualitative experiences from the same intervention). In such cases, we noted the overlap in the evidence tables and synthesized the studies narratively, without double counting participants in the interpretation of results.

### 2.3. Data Extraction and Synthesis

For each eligible study, data were extracted using a standardized form: author, year, country, study design, population, type of loss, relationship to deceased, time since loss, sample size, intervention name, delivery mode, duration and dose, comparator, primary and secondary measures, main findings, other findings, and adverse events. Given the heterogeneity of study designs, interventions, and outcomes, findings were synthesized narratively and summarized in evidence tables. No meta-analysis was planned.

### 2.4. Quality Appraisal

The methodological quality of the included studies was appraised using a pragmatic approach adapted to the aims of this systematized narrative review. Given the marked heterogeneity of study designs included in this review (RCTs, quasi-experimental, pre–post, and qualitative studies), a simplified appraisal strategy was adopted to ensure consistency across the analysis. Therefore, each study was evaluated along two dimensions: (a) effect direction (i.e., whether the intervention showed improvements [↑], no effect [↔], or deterioration [↓] on grief-related or secondary outcomes), and (b) an overall quality rating based on study design, sample size, follow-up, and appropriateness of outcome measures. The quality rating was summarized using a traffic-light system:

- Green = higher-quality evidence (well-designed RCTs or large controlled studies with grief-specific outcomes and adequate follow-up),
- Yellow = moderate-quality evidence (pilot trials, quasi-experimental, qualitative, or controlled studies without grief-specific outcomes),
- Red = low-quality evidence (small uncontrolled studies, descriptive reports, or studies with major methodological flaws).

Each study was independently evaluated by two reviewers using the three-level traffic-light system based on predefined methodological criteria. To ensure the reliability of the traffic-light classification, inter-rater agreement between the two independent reviewers was assessed using Cohen’s unweighted kappa. The unweighted version was selected because the traffic-light categories represent nominal classifications without an inherent quantitative distance. Inter-rater agreement was used exclusively to assess the reliability of the appraisal procedure and does not imply validation of the traffic-light system as a formal quality assessment tool. Any disagreement was subsequently resolved through discussion.

In accordance with guidance on synthesis without meta-analysis, a pragmatic appraisal strategy was adopted to summarize heterogeneous evidence using direction-of-effect coding and transparent study descriptors, rather than applying formal risk-of-bias tools across highly diverse designs. The traffic-light system was used as a visual aid to support clarity and interpretability across studies and should be understood as an integrative summary of methodological considerations rather than a substitute for structured risk-of-bias assessment [35]. The operational criteria used to assign the traffic-light ratings are reported in Appendix A (Table A1). The use of a traffic-light system reflects established evidence-grading principles, whereby complex and continuous judgments about methodological quality are summarized into discrete categories, while acknowledging the inherent arbitrariness of such classifications [36].

### 2.5. Study Selection Process

The database search (2000–2025) yielded a total of 389 records (PubMed *n* = 83; PsycINFO *n* = 96; Scopus *n* = 54; Web of Science *n* = 156). After duplicate removal, 105 unique records remained for title and abstract screening.

**Figure 1:**
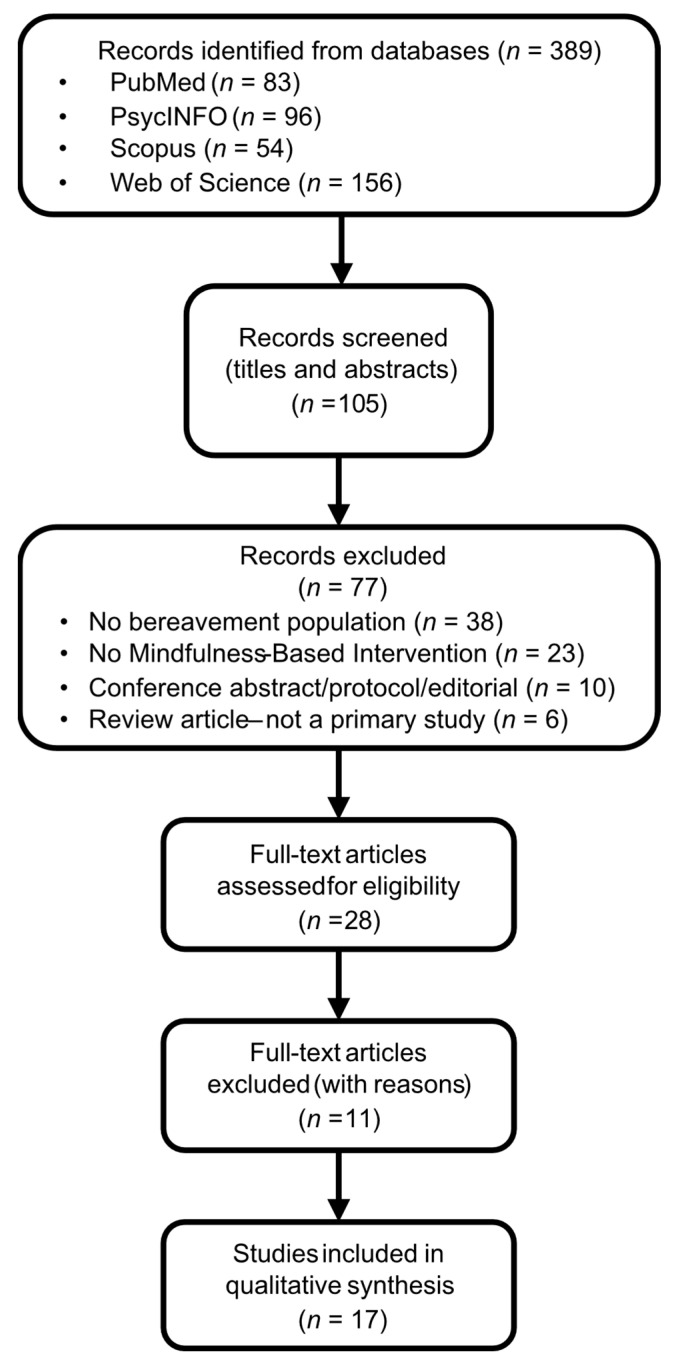
PRISMA-style flow diagram of the study selection process.

Given the variability in intervention protocols, grief populations, and measured outcomes, a narrative synthesis was considered more appropriate than a quantitative meta-analysis. This approach enables a structured evaluation of methodological quality while allowing for theoretical integration across diverse study contexts. The study selection process is depicted in a PRISMA-style flow diagram adapted for narrative reviews (Figure 1). Eleven full-text articles were excluded after eligibility assessment, and the complete list of these excluded studies together with the specific reasons for exclusion is provided in Appendix B—Table A2.

## 3. Results

### 3.1. Characteristics of the Included Studies

**Figure 2:**
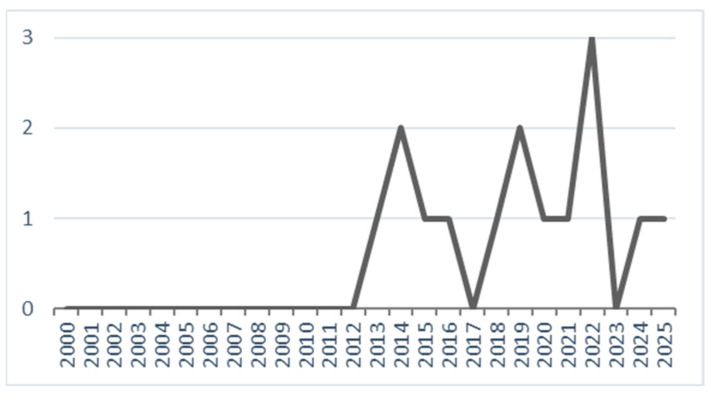
Chronological distribution of the studies.

**Figure 3:**
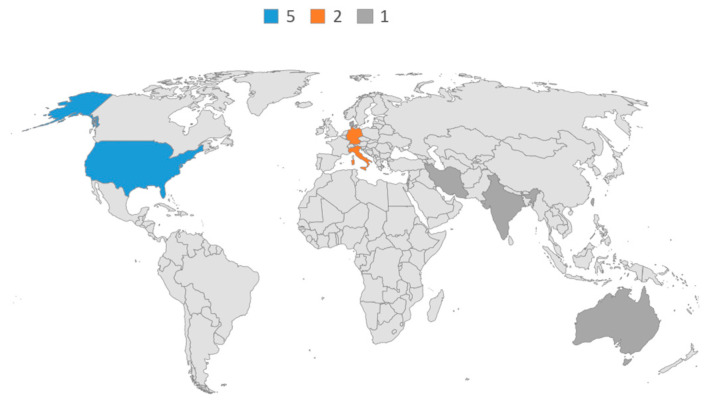
Distribution of included studies by country of origin. Numbers indicate the total number of included studies per country; light blue, orange, and dark grey are used for visual distinction of the country distributions.

A total of 17 studies met the eligibility criteria, published between 2013 and 2025 (see Table 1). When considering only unique studies (excluding secondary analyses or publications based on the same participant cohort), the chronological distribution shows an emerging but still limited evidence base. The first eligible study was published in 2013, followed by a gradual increase with peaks in 2014 (2 studies), 2019 (2 studies), and 2022 (3 studies). In recent years, research has continued steadily, with single studies published in 2024 and 2025 (see Figure 2). Overall, the evidence remains sparse, with no more than three new studies published in a single year. The majority of studies were conducted in the United States (*n* = 5), followed by Germany (*n* = 2) and Italy (*n* = 2). Single studies were carried out in Australia, Denmark, India, Iran, Israel, and Taiwan (see Figure 3). This distribution indicates that the current evidence base is concentrated in Western countries, particularly the United States, while research from Asia and the Middle East remains limited to isolated contributions.

Moreover, studies conducted in Western countries often report samples that are predominantly White and with higher levels of education [38,42,51,54]. However, studies from Iran and India highlight different socioeconomic and cultural contexts, focusing on women from public hospitals or rural, low-income backgrounds, sometimes with low literacy rates [46,48].

### 3.2. Research Design

The analysis of research designs across the included sources indicates that 15 studies involved primary data collection, employing either quantitative or qualitative methodologies. Two additional publications [44,53] were based on secondary analyses or subsamples drawn from previously published primary studies. Specifically, Ref. [43] reanalyzed data from the randomized controlled trial reported in [42], while the qualitative study by [52] was conducted on a subsample of participants from the quasi-experimental study reported in [51]. Accordingly, these two sources were retained in the total count of 17 included publications, but they do not represent distinct primary studies in terms of original data acquisition.

As summarized in Table 1, the 17 included studies employed a wide range of research designs. Only one study adopted a fully powered and methodologically rigorous randomized controlled trial (RCT) design, directly comparing Cognitive–Behavioral Therapy (CBT) with Mindfulness-Based Cognitive Therapy (MBCT) for PGD [38]. Two additional RCTs, conducted in specific bereavement-related populations, evaluated MBIs: one focused on women following early pregnancy loss [46], and the other tested Existential Behavioral Therapy (EBT) among informal caregivers of patients receiving palliative care [45]. Several studies employed quasi-experimental or non-randomized controlled designs, typically incorporating comparison groups without full random allocation. These included a controlled trial comparing Mindfulness Training (MT) with Progressive Muscle Relaxation (PMR) and a waitlist control group in widowed individuals [42], with secondary outcome analyses reported separately [43]. Other non-randomized studies evaluated MBCT in older bereaved adults [47], examined a brief and intensive MBI for suicide-loss survivors [50], and applied a non-equivalent comparison group design to assess a mindfulness-based retreat for bereaved parents [51].

A substantial proportion of the evidence base consisted of single-group pre–post de-signs, primarily pilot or feasibility studies assessing changes before and after mindful-ness-based programs without the inclusion of a control group. These included a retrospective cohort analysis of a mind–body group intervention for mothers following stillbirth [37], investigations of MBCT in bereaved individuals incorporating functional neuroimaging methods [39], an uncontrolled pilot study of MBSR for victims of gun violence [41], a retrospective chart review evaluating the ATTEND model in traumatic grief counseling [53], a pilot study of a culturally adapted MBI for perinatal bereavement in India [48], and a feasibility pilot study of residential retreats for suicide-loss survivors [49].

One study relied on cross-sectional baseline data only [54], while two studies employed qualitative methodologies to explore participants’ lived experiences. These included an analysis of perceived helpful factors within Existential Behavioral Therapy (EBT) [44] and a thematic analysis of bereaved parents’ perceptions of a mindfulness-based retreat [52].

Overall, this distribution indicates a predominant focus on intervention research, with most studies designed to evaluate the feasibility, acceptability, or preliminary efficacy of mindfulness-based approaches for bereavement. While RCTs provide higher-level evidence, the majority of available studies remain exploratory or quasi-experimental, underscoring the need for more controlled trials with larger and more diverse samples.

### 3.3. Type of Loss

The included studies varied in the type of bereavement targeted, although several re-ports did not specify this information in detail. Among those that did, the most common categories were suicide-related deaths (e.g., [49,51]), perinatal or stillbirth loss (e.g., [41,42,47]), and illness-related deaths, often in palliative care or cancer contexts (e.g., [44,45]). Traumatic deaths, such as gun violence, were specifically addressed in [41], while medical fatalities and unintentional injuries were included in the randomized trial by [38]. In some studies, detailed percentages were provided: for instance, Ref. [38] reported that 64% of losses were due to medical causes, 28% to suicide, and 8% to unintentional injuries. Other investigations, particularly those employing pre–post or qualitative methodologies (e.g., [39,40]), described participants as bereaved adults but did not report the specific type of death. Overall, the distribution suggests that suicide and perinatal loss are the most frequently represented causes of bereavement, with smaller but important contributions from illness, traumatic, and medical/unintentional deaths. The lack of systematic reporting of the cause of death in several studies limits the possibility to compare intervention effects across different types of bereavement.

### 3.4. Relationship with the Deceased

Information on participants’ relationship to the deceased was inconsistently reported across the included studies. When specified, the most frequently represented categories were spousal/partner loss and child loss, followed by parental loss and sibling loss. For example, Ref. [38] reported that participants were primarily bereaved of a parent (52%), child (22%), or partner (14%), with smaller proportions losing siblings (10%) or grandparents (2%). Similarly, Refs. [49,50] described samples that included partners, children, siblings, and parents bereaved by suicide, whereas [42] focused exclusively on widowed adults. Other studies targeted narrower categories: Refs. [37,48] included only bereaved mothers after stillbirth, while [46] investigated women following early pregnancy loss. Thieleman, Cacciatore, and colleagues (2014; 2020; 2022) focused specifically on bereaved parents, often in the context of traumatic loss. By contrast, several studies (e.g., [39,44,45,47]) reported that participants were “bereaved adults” but did not consistently specify their relationship to the deceased. Taken together, the evidence base is dominated by studies involving parents and partners, with relatively fewer studies addressing sibling, grandparent, or more extended kinship losses. The lack of systematic reporting of relationship categories across studies limits the possibility of assessing whether intervention effects differ according to the type of bond with the deceased.

### 3.5. Time Since Loss

The interval between bereavement and participation in the intervention was highly variable across the included studies. Some trials focused on recent loss, such as [37], where women bereaved by stillbirth were enrolled on average less than two months after the event (M = 1.79 months), or [39,40], which included participants bereaved within 6 months to 4 years. Similarly, Ref. [46] targeted women shortly after early pregnancy loss, although exact timing was not reported. Other studies involved longer-term bereavement. For instance, Refs. [42,43] included widowed adults approximately 14 months post-loss (M = 14.46 months), Ref. [47] recruited bereaved partners around four years after loss, and [44,45] reported mean times since loss of over one year. Refs. [49,50] described heterogeneous samples bereaved by suicide with median or mean durations of more than two years (median ~840 days, range 68–11.285 days), while [51,52,53] included parents bereaved on average between 1.7 and 5 years earlier. Several studies did not clearly report time since loss, limiting comparability (e.g., [41,48]). Overall, the evidence base includes both early interventions (within weeks or months of the death) and long-term interventions targeting bereavement persisting for several years. This variability reflects the diverse contexts in which mindfulness-based approaches have been tested, but it also complicates the possibility to evaluate timing as a moderator of intervention effects.

### 3.6. Sample Size

The sample sizes for MBI groups range from as few as 12 completers [47] and 19–20 in within-subject designs ([39,40], respectively) to larger groups of 72–74 ([37,54], respectively) and 97 [46]. Randomized controlled trials, such as [38], aimed for 50 participants in the MBCT arm, while [42,43] had 37 assigned to Mindfulness Training (MT), with about 30 completing the intervention. Many studies explicitly state their pilot nature (e.g., [41,47,48,49]), and thus inherently feature smaller sample sizes. These studies serve to explore feasibility and preliminary efficacy, paving the way for larger trials. Small sample sizes often lead to in-sufficient statistical power to detect significant effects, particularly for subtle changes or when dealing with high variability in grief experiences [47,54]. Some studies mention aiming for specific sample sizes based on power calculations (e.g., [38,40]) but do not always achieve them. A recurring limitation is that the samples are often small, self-selected, and homogeneous (e.g., predominantly White, female, or highly educated), limiting the generalizability of findings to broader, more di-verse bereaved populations [41,42,43,48,51]. This limitation is particularly critical in trauma contexts such as gun violence victimization, where the demographic characteristics of the affected population may differ substantially from those of the study participants [41]. Small samples can make it difficult to differentiate between intervention effects and other factors (e.g., natural recovery, social support, or regression to the mean) [38,41]. The need for replication in larger, more diverse, and rigorously controlled trials is a consistent recommendation across the sources [47,48,50]. In summary, while the existing body of literature provides encouraging preliminary evidence for the benefits of MBIs in various bereaved populations, it also underscores the need for larger, well-powered randomized controlled trials with more diverse samples to confirm these findings and enhance their generalizability and clinical applicability.

### 3.7. Intervention

#### 3.7.1. Foundational Mindfulness-Based Programs (MBSR/MBCT and Their Adaptations)

These are typically structured, often 8-week, group-based programs that emphasize both formal (e.g., meditation) and informal (e.g., daily life awareness) mindfulness practices. MBSR is an 8-week evidence-based program designed to train individuals in non-judgmental reactivity to sensory events by focusing attention on somatic sensations like breath and body [41]. It provides intensive training that often includes practices such as body scan, mindful awareness of breath, sitting meditation, Hatha Yoga, emotional regulation, and compassion for others [41]. Participants are given daily assignments and encouraged to integrate mindfulness into daily life [41,46]. The goal is to be present in the moment without excessive worry about the future or past [46]. Sessions may also cover stress and body reactions, thought-emotion-body senses-behavior relationships, short concentration exercises (e.g., Three-Minute Breathing Space), mindful walking, conscious mind interactions, and “mountain meditation” [46]. This intervention was used in a pilot study for victims of gun violence [41] and for women with early pregnancy loss [46]. A shortened, culturally adapted five-week MBI was developed from the MBSR curriculum for women after stillbirth in rural India. It included mindfulness skills for daily life and psychoeducation on stillbirth risk factors, delivered by trained local nurses [48].

MBCT is an 8-week group-based clinical intervention that integrates elements of Cognitive-Behavioral Therapy (CBT) with systematic mindfulness meditation training [47]. Its aim is to teach participants to become more aware of and relate differently to their thoughts, feelings, and bodily sensations [47]. Key practices involve learning to accept in-tense emotional distress non-judgmentally, detaching attention from the content of automatic thoughts, and regulating attention back to present-moment experiences like the breath or bodily sensations [47]. MBCT also addresses unhelpful appraisals, which are considered crucial in maintaining PGD [38]. It typically includes guided meditations (such as body scans, sitting meditation, compassion meditation, and yoga), experiential exercises, discussions, and daily home practice. For bereaved individuals, specific adaptations like an additional introductory session on “acknowledgment of grief and theory of psycho-physical reactions to loss” might be included [39]. For elderly participants, session duration might be slightly reduced, and the focus of psycho-educational parts adapted [47]. MBCT has been applied in studies for PGD [38], for bereaved individuals (with fMRI analysis) [39,40], and for elderly bereaved people with loss-related distress [47].

#### 3.7.2. Broader Mindfulness Training and Mind-Body Interventions

These interventions incorporate mindfulness as a key component but might not strictly adhere to the MBSR/MBCT manuals, often blending mindfulness with other therapeutic elements or using a more general “Mindfulness Training” (MT) label.

This latter type of intervention typically involves a systematic practice of focusing attention on present-moment experiences, emotions, and thoughts from an open, nonreactive, and nonjudgmental perspective [42,43]. Often based on programs like the UCLA Mindful Awareness Practices Level 1 course, it includes sessions on introduction to mindfulness, listening, working with pain/difficult emotions/thoughts, mindful interactions, and cultivating positive emotions (e.g., loving kindness) [42,43]. In-session practices can range from 10–25 min, with daily home practice of guided meditation (e.g., 5–19 min) [42,43]. The goal is to hone focused and receptive attention to reduce the automaticity of habits of mind like yearning and rumination, leading to “decentering” (observing thoughts/feelings as temporary mental phenomena) [42]. MTs were employed for bereavement-related grief in widow(er)s [42,43].

Mind-Body Group Therapy (MBGT). MBGT is a holistic therapeutic approach that combines techniques to enhance the connection between mental and physical health [37]. It includes practices such as relaxation techniques, mindfulness, cognitive behavioral strategies, and psychoeducation to reduce stress, improve emotional resilience, and alleviate psychological or physical symptoms [37]. The protocol, adapted from an existing model for infertility, involves eight group sessions and targets populations like women who experienced stillbirth [37].

Existential Behavioural Therapy (EBT). EBT is a group intervention, typically comprising six weekly meetings (22 h in total), designed to support informal caregivers of palliative patients during caregiving and bereavement [44]. Mindfulness training is a core element, integrated into the “third wave” of behavioral therapy alongside existential psychology [44]. Mindfulness, defined as focusing attention on the present experience with a nonjudgmental, accepting attitude, is practiced formally (e.g., following one’s breath, noticing and letting go of thoughts/feelings/sensations) in every session (at least 15 min) and encouraged at home (formally twice a day for 10 min, and informally through daily activities) [44,45]. This practice aims to help individuals (e.g., caregivers of palliative patients) distance themselves from ruminative thoughts and painful feelings [44].

#### 3.7.3. Retreat-Based Mindfulness Interventions

These interventions are typically more intensive and shorter in duration, often offered in a retreat format, and frequently incorporate elements of self-compassion.

Mindfulness-based weekend retreats (Panta Rhei). These are short, intensive experiential retreats (16 h of practice over two days), based on principles from MBSR, MBCT, and Mindful Self-Compassion (MSC) [49,50]. They are specifically tailored for people bereaved by suicide, focusing on addressing grief pain and facilitating “letting go” [49,50]. Practices include Raisin Meditation, Body Scan, Breath Meditation, exercises like “How would I treat a friend?” and “Why am I here? What do I need?”, meditation on a compassionate friend, loving-kindness (Metta) meditation, kindness practice, mindful walking, mindful yoga, and mindfulness of sounds, colors, and smells [49,50]. The intervention emphasizes non-judgmental awareness, self-compassion, and acceptance, often within a supportive group setting with moments for silence and interaction [49,50].

Grief-focused mindfulness-based retreat (Selah). A 4-day retreat for bereaved parents that combines mindfulness practices with psychoeducation about child death [51,52]. It aims to help parents understand their experiences within a broader, often invalidating social context, fostering interpersonal and communal aspects of healing [51,52]. The retreat focuses on reducing psychological distress (trauma, anxiety, depression) and increasing well-being (mindfulness and self-compassion) [51]. Participants learn to bring an open and accepting attitude to their experiences, with reported benefits including a deepened sense of awareness through guided meditations, grief-related exercises, mindful movement, and art [52].

ATTEND model. This model is a MBI where clinicians are specifically trained in and encouraged to practice mindfulness (e.g., meditation, various styles of yoga, mindful movement, speech, and actions) and self-care [51,53]. The model’s six elements are Attunement, Trust, Therapeutic touch, Egalitarianism, Nuance, and Death education [53]. Clinician mindfulness is central to “Attunement,” fostering empathy, compassion, and connection. The goals are to increase emotional tolerance and awareness in clients by modelling non-judgmental acceptance. Clients may be taught specific mindfulness practices like 15-min breath exercises, awareness journaling, and nature walks. The model promotes individualized care rather than a rigid protocol [53].

In summary, the evidence base reflects a broad spectrum of MBIs adapted for bereavement populations. Foundational and manualized programs such as MBSR and MBCT remain the most widely applied, often delivered in standardized 8-week formats, while other studies tested shortened or culturally adapted versions tailored to specific contexts (e.g., stillbirth in India, pregnancy loss in Iran). Beyond these, several trials evaluated broader mindfulness training programs and integrative approaches such as Mind-Body Group Therapy (MBGT) and Existential Behavioral Therapy (EBT), embedding mindfulness practice within wider therapeutic frameworks. A distinct line of research has focused on retreat-based interventions, including the Panta Rhei model for suicide bereavement and the Selah retreats for bereaved parents, which combine intensive mindfulness and self-compassion practices in supportive group settings. Finally, innovative models such as the ATTEND framework emphasize clinician mindfulness as a relational vehicle for attunement and compassion in bereavement care. Taken together, these interventions illustrate the flexibility and adaptability of mindfulness-based approaches, spanning from standardized protocols to culturally specific adaptations and retreat formats. However, the diversity of programs and variability in their implementation complicate direct comparisons and underscores the need for future research to clarify which formats, components, and delivery modes are most effective for different bereavement populations.

### 3.8. Delivery Mode

Across the 17 included studies, the delivery of MBIs for bereavement was heterogeneous.

Group-based, in-person interventions were most common, including MBCT, MBSR, MBGT, MT, and EBT [37,39,40,42,43,44,45,46,47]. Retreat-based formats, such as Panta Rhei and the Selah retreats, offered intensive short-term group experiences for suicide-bereaved adults and bereaved parents [49,50,51,52]. Online delivery was less frequent but feasible, as shown by [41] with MBSR delivered via videoconference. Individual interventions were relatively rare: MBCT adapted for prolonged grief dis-order was administered individually [38], while the ATTEND model provided individualized therapy guided by trained clinicians [53].

In summary, while in-person group interventions predominate, emerging evidence highlights the feasibility of online programs and the potential of individualized approaches, with retreat-based models adding further diversity to available formats.

### 3.9. Duration and Dose

The duration and intensity of interventions varied considerably. Most manualized programs followed standardized structures of 6–8 weekly sessions, each lasting 2–2.5 h [39,40,42,43,46,47]. MBGT also comprised eight weekly sessions [37]. EBT was delivered in six group sessions of approximately 3.5 h [44,45]. Shorter and adapted interventions included the five-week culturally tailored MBSR for stillbirth in India [48]. More intensive formats were represented by the Panta Rhei weekend retreat (16 h across two days) [49,50] and the Selah retreats (four consecutive days) [51,52]. Individualized formats varied: MBCT for PGD involved 11 weekly 90-min sessions [38], while the ATTEND model extended over an average of 19 weeks with flexible session structures [53].

Several studies encouraged daily home practice ranging from 15–40 min, often supported by guided audio or written materials (e.g., [39,40,48,49,50]). Retreat formats, by contrast, concentrated practice into immersive schedules with multiple daily sessions.

In sum, dose ranged from brief 5-week courses to multi-month individual therapy and multi-day retreats, with most studies converging on the 6–8 week, 2-h weekly group session as the dominant structure, usually reinforced by regular home practice.

### 3.10. Comparator

The comparators employed across the included studies were heterogeneous, reflecting both the exploratory nature of much of the research and the diversity of bereavement populations.

Only a minority of studies incorporated active comparators. Ref. [38] directly compared MBCT adapted for grief with grief-focused CBT, demonstrating superior outcomes for the CBT arm at 6-month follow-up. Refs. [42,43] contrasted Mindfulness Training (MT) with Progressive Muscle Relaxation (PMR) and a wait-list control, showing that both MT and PMR reduced grief-related distress, though PMR out-performed MT for some outcomes. Several studies used passive control groups. Ref. [47] employed a wait-list design for bereaved partners, while [52] compared participants attending the Panta Rhei retreat with a non-intervention control group recruited from an online suicide-bereavement forum. Ref. [51] also used a passive comparison group of bereaved parents who did not attend the Selah retreat.

In contrast, many investigations were conducted as single-arm pre–post studies without a control condition, such as [42,47,48,49,51,54]. These designs provide useful feasibility and acceptability data but limit causal inference.

Finally, some studies employed qualitative or uncontrolled frameworks [44,52], where no comparator was applicable.

In summary, while a few well-designed trials included active or passive comparators, the majority of studies relied on uncontrolled pre–post designs. This imbalance highlights the need for future research employing rigorous randomized comparisons with active control conditions to better isolate the specific effects of mindfulness-based interventions in bereavement.

### 3.11. Primary Grief Outcome Measure

The use of grief-specific outcome measures was inconsistent across the included studies.

Among the randomized controlled trials, Ref. [38] employed the Prolonged Grief Disorder scale (PG-13) [29,55] and the Grief Cognitions Questionnaire (GCQ) [56] as primary measures. Ref. [42] used the Inventory of Complicated Grief—Revised (ICG-R) [57,58], complemented by yearning (YSL) [59] and grief rumination (UGRS), whereas [47] also relied on the ICG-R. Refs. [39,40] administered the Texas Revised Inventory of Grief (TRIG) [60] to evaluate pre–post changes in bereaved adults. Ref. [48] employed the Perinatal Grief Scale (PGS) [61,62] in their culturally adapted intervention for women after stillbirth. Ref. [41] used the Inventory of Complicated Grief (ICG) in a sample of adults bereaved by gun violence, though high dropout limited analyses.

By contrast, several studies did not include a direct grief-specific measure. These included [37], which conceptualized stillbirth grief indirectly and focused on depressive and trauma-related outcomes [46], which assessed psychological distress using the DASS-21; and [44,45], which relied on psychological well-being, quality of life, and mindfulness outcomes. Retreat-based interventions such as Panta Rhei [49,50] and the Selah retreats [51,52] also did not include standardized grief measures, instead evaluating psychological distress, mindfulness, and self-compassion. The ATTEND model was assessed primarily through trauma- and depression-related indicators rather than grief-specific scales [53].

The grief-related outcome measures employed across the included studies are sup-ported by established psychometric validity and reliability, although their heterogeneous use limits direct comparability of findings. The PG-13 demonstrates strong internal consistency (α = 0.89) and alignment with ICD-11 diagnostic criteria [38], while the ICG/ICG-R shows excellent internal consistency (α ≈ 0.94), good test–retest reliability (r ≈ 0.80), and solid criterion validity [42]. The PGS also demonstrates good reliability (α = 0.89) in perinatal populations [48]. Measures targeting grief-related processes, such as the YSL and the UGRS, exhibit excellent internal consistency (α = 0.85–0.97) and strong discriminant validity from depression and anxiety [42], while the TRIG remains a widely used and reliable measure of grief severity (α = 0.89–0.94) [39,40].

In summary, less than half of the included studies employed validated grief-specific measures (e.g., PG-13, ICG, ICG-R, TRIG, PGS), while the remainder relied on indirect indicators such as depression, anxiety, trauma, or quality of life. This methodological gap reduces the comparability of findings and limits the possibility to draw firm conclusions about the efficacy of mindfulness-based interventions in alleviating grief itself.

### 3.12. Secondary Outcome Measures

Beyond grief-specific assessments, most of the included studies incorporated a wide range of secondary measures addressing broader domains of psychological distress and well-being [37].

Depression was frequently evaluated, using instruments such as the Beck Depression Inventory-II [38,41,47,63], the Edinburgh Postnatal Depression Scale [37,64], the Tai-wan Depression Scale [39,40,65], or the depression subscale of the DASS-21 [46,66]. Anxiety was similarly assessed across several trials, most often with the Beck Anxiety Inventory [38,67], the Generalized Anxiety Disorder Scale [39,40,68], or the anxiety subscale of the DASS-21 [46,66]. The Hopkins Symptom Checklist (HSCL) [69], in its 10- and 25-item versions (HSCL-10 and HSCL-25), was also employed as a measure of anxiety and depressive symptomatology [48,51,53].

Post-traumatic stress symptoms were also a central focus, measured with tools such as the PTSD Checklist for DSM-5 [41,70,71], the Trauma Symptom Checklist-40 [41,72], the Harvard Trauma Questionnaire [43,47,73], and the Impact of Event Scale–Revised [51,53,74], while coping responses to trauma were captured by the Perceived Ability to Cope with Trauma Scale, specifically its flexibility subscale (PACT-flexibility) [43,75]. Other studies focused on stress and emotion regulation, using the Perceived Stress Scale [43,76] and the Difficulties in Emotion Regulation Scale [39,40,77]).

Consistent with the mindfulness focus of these interventions, nearly all programs included validated instruments to capture dispositional mindfulness, most prominently the Five Facet Mindfulness Questionnaire [78] (e.g., [39,40,41,48,49,50,51]), but also the Cognitive and Affective Mindfulness Scale–Revised [45,79] and the Mindful Attention Awareness Scale [43,54,80]. Self-compassion was another recurrent domain, measured through the Self-Compassion Scale in either its full or short form [49,50,51,81].

Finally, several studies investigated broader indicators of well-being and functioning, including the Satisfaction With Life Scale [41,43,45,48,82], the WHO Quality of Life-BREF [38,45,83,84], and the Schedule for the Evaluation of Individual Quality of Life [45,85]. A smaller number of investigations extended to additional domains such as sleep quality (Pittsburgh Sleep Quality Index—PSQI) [43,54,86], loneliness (UCLA Loneliness Scale) [43,87], religious coping (Brief RCOPE) [48,88], social support [48], and even cognitive performance tasks [39,59].

With respect to the validity and reliability of the principal scales not specifically de-signed for grief assessment, the PCL-5, used to assess post-traumatic stress symptoms in bereaved populations, demonstrates excellent internal consistency (α ranging from 0.91 to 0.93) [37,41]. Similarly, the EPDS and the STAI-S, applied in perinatal bereavement contexts, show strong internal reliability (α = 0.93 and α = 0.89, respectively) [37]. The FFMQ, widely used to assess dispositional mindfulness, is also well validated, with high overall reliability in both meditator (α ≈ 0.95) and non-meditator samples (α ≈ 0.86) [39,49].

This heterogeneity of secondary outcome measures illustrates the multidimensional scope of mindfulness-based interventions in bereavement, but also highlights the challenge of synthesizing findings across such diverse constructs.

### 3.13. Descriptive Synthesis

Across the 17 included studies, mindfulness-based interventions for bereavement were applied in highly heterogeneous ways, reflecting differences in populations, study designs, intervention formats, and outcome measures. This heterogeneity precludes quantitative pooling but allows for an integrative narrative overview. Most programs were delivered as structured group interventions based on established protocols such as MBCT or MBSR, while others employed retreat-based or individualized formats. Study designs ranged from randomized controlled trials to non-randomized controlled studies, single-arm pre–post designs, and qualitative investigations. Sample sizes varied considerably, from fewer than 20 participants in small pilot trials to nearly 150 in larger non-randomized studies.

The assessment of outcomes was equally diverse. While some studies employed validated grief-specific measures (e.g., PG-13, ICG, ICG-R, TRIG, PGS), many relied instead on indirect indicators such as depression, anxiety, post-traumatic stress, mindfulness, self-compassion, and quality of life. The uneven use of grief-specific instruments complicates comparability across studies and constrains the possibility to draw firm conclusions about the direct impact of mindfulness on grief per se.

Overall, the evidence suggests that mindfulness-based interventions are generally feasible, well tolerated, and potentially beneficial across multiple domains of psychological adjustment in bereavement. However, the heterogeneity of designs, the predominance of small and uncontrolled studies, and the inconsistent use of grief-specific outcomes underscore the need for cautious interpretation. The following sections present the main intervention outcomes and adverse events reported in the included studies.

### 3.14. Main Outcomes

#### 3.14.1. Bereavement Related Outcomes

Among the 17 included studies, fewer than half employed direct grief-specific measures, while two relied on qualitative assessments of grief-related experiences (see Table 2).

The strongest evidence derives from the randomized controlled trial by [38], where MBCT adapted for grief significantly reduced prolonged grief disorder symptoms (PG-13). Both MBCT and grief-focused CBT showed large within-group improvements, though CBT proved superior at six-month follow-up, particularly in reducing maladaptive grief-related cognitions (GCQ).

Other controlled studies provided supportive findings. Ref. [42] reported significant reductions in complicated grief (ICG-R), yearning (YSL), and grief-related rumination (UGRS) following both Mindfulness Training (MT) and Progressive Muscle Relaxation (PMR), with PMR showing stronger effects than MT and wait-list. In contrast, the secondary analysis [43] did not include grief-specific outcomes, limiting its relevance to bereavement per se.

Uncontrolled pre–post studies further suggested potential benefits. Refs. [39,40] observed robust reductions in grief (TRIG) among bereaved adults after MBCT, complemented by neuroimaging findings linking grief improvement to changes in neural connectivity supporting emotion regulation. Similarly, Ref. [48] found significant decreases in perinatal grief (PGS) in Indian women following a culturally adapted five-week MBSR program.

Evidence in contexts of traumatic loss was weaker. Ref. [41] documented a 23% reduction in complicated grief (ICG) after MBSR for survivors of gun violence, though the effect did not remain significant after correction for multiple testing due to small sample size and attrition. Ref. [47] also reported no significant effects of MBCT on grief (ICG-R) compared with waitlist, in a small pilot with high dropout.

Qualitative studies provided convergent insights. Ref. [44] highlighted acceptance, reduced rumination, and reorientation as perceived benefits of mindfulness, while [52] emphasized enhanced self-compassion, continuing bonds, and validation of grief in a supportive retreat setting, albeit with some participants experiencing temporary distress when exposed to others’ traumatic narratives.

In summary, the available evidence suggests that mindfulness-based interventions can alleviate grief symptoms, with consistent improvements observed in validated scales such as the PG-13, ICG-R, TRIG, and PGS. Qualitative data reinforce these findings by illustrating processes of acceptance, reduced rumination, and strengthened continuing bonds. Nonetheless, the overall strength of evidence remains limited by the small number of RCTs, modest sample sizes, and inconsistent use of grief-specific outcomes.

#### 3.14.2. Other Findings

Several studies reported additional results that, although not designated as primary outcomes, provide valuable insights into grief processes and their modulation through mindfulness. In the trial by [42], reductions in yearning and grief-related rumination paralleled the decline in grief severity, suggesting that mindfulness training may operate by reducing maladaptive cognitive processes that sustain bereavement distress. Similarly, Refs. [39,40] found that increases in dispositional mindfulness were negatively correlated with grief severity (TRIG), indicating that enhanced mindful awareness may buffer against intrusive grief-related thoughts and emotions.

Studies also identified neurocognitive correlates of change. Ref. [39] demonstrated that improvements in grief scores were associated with reduced activation in the posterior cingulate cortex and thalamus, while [40] highlighted changes in cortico-subcortical connectivity linked to emotional regulation. These findings suggest that the beneficial effects of MBCT on grief may be mediated by neural mechanisms supporting attentional control and affective regulation.

In contexts of traumatic loss, Ref. [41] observed that gains in mindfulness predicted greater reductions in complicated grief, even though overall grief score changes did not remain significant after correction. This underscores the potential role of mindfulness as a mediator of grief adaptation, even in highly challenging bereavements such as gun violence.

Qualitative evidence further deepens this picture. Ref. [44] reported that participants described mindfulness and acceptance as tools to counter ruminative thinking and to foster reorientation after loss, while [52] emphasized how retreat participation supported continuing bonds, communal validation of grief, and improved emotional regulation. At the same time, some participants described transient distress when exposed to others’ traumatic narratives, highlighting the ambivalence of group processes in bereavement.

Taken together, these findings suggest that mindfulness-based interventions may influence grief not only by reducing symptom severity but also by addressing underlying processes such as yearning, rumination, emotional regulation, and continuing bonds. The convergence of quantitative and qualitative evidence points toward a multifaceted role of mindfulness in reshaping the way bereaved individuals relate to their loss.

### 3.15. Acceptability and Safety

Across the included studies, mindfulness-based interventions for bereavement were generally considered safe and acceptable, with very few adverse events reported. No study described serious harms attributable to the intervention. However, some participants noted transient emotional discomfort. For instance, in the Selah retreats, exposure to others’ traumatic narratives occasionally intensified grief-related emotions, a phenomenon described as cathartic rather than harmful when contained within a safe and supportive group environment [51,52]. Similarly, participants in the Panta Rhei retreats sometimes reported emotional intensity during practices, though this was interpreted as part of the grief process rather than an adverse effect [49,50].

Retention varied widely across studies and constituted a recurrent methodological challenge. The large RCT by [38] retained only 60% of participants at 6-month follow-up, limiting the strength of conclusions regarding long-term efficacy. Ref. [47] reported that only 36% of eligible participants ultimately completed the MBCT intervention, reflecting difficulties in engaging bereaved individuals in longer protocols. In contrast, retreat-based programs such as Panta Rhei showed relatively high adherence, with most participants completing the intensive weekend format and reporting strong acceptability [49,50]. Similarly, Ref. [48] observed adherence rates close to 90% in the culturally adapted MBSR program for stillbirth in India, facilitated by shorter duration and local delivery.

Overall, dropout rates tended to be higher in multi-week clinical trials than in brief, retreat-based, or culturally adapted programs. This pattern suggests that while mindful-ness interventions are generally acceptable, their feasibility may depend on program length, intensity, and contextual tailoring to bereaved populations.

In summary, MBIs for bereavement appear to be safe, with no evidence of intervention-related harm, and generally acceptable to participants. Nonetheless, in the context of suicide bereavement, facilitators should remain attentive to the risk of temporary emotional intensification, particularly during group sharing, which may require additional containment strategies. Feasibility also depends on program format: attrition tends to be higher in longer clinical trials, whereas brief, retreat-based, or culturally adapted programs show consistently higher adherence and participant satisfaction.

### 3.16. Quality of Included Studies

Inter-rater agreement for the traffic-light classification was substantial (Cohen’s unweighted κ = 0.64). Disagreement occurred in only one of the 17 studies evaluated (94% raw agreement). The kappa coefficient should be interpreted in light of the small number of ratings and the skewed marginal distributions across categories, conditions that attenuate κ values despite high observed agreement. In the present case, the obtained κ approached the maximum possible value given the observed marginal frequencies. The methodological appraisal showed that only one study, the randomized controlled trial by [38], met criteria for high-quality evidence (Green rating), as it combined a rigorous randomized design, an adequate sample size, and the use of grief-specific outcome measures. All of the remaining 16 studies were classified as moderate quality (Yellow rating). This was due either to limitations in study design (e.g., non-randomized controlled trials, pre–post pilots, qualitative investigations, or secondary analyses) or to the absence of grief-specific outcome measures, which led to systematic downgrading irrespective of methodological rigor. No study was classified as low quality (Red rating). Table 3 provides a synthesis of effect directions across grief-related and secondary outcomes, together with the overall quality rating for each study based on the traffic-light appraisal system.

Across the evidence base, several recurring limitations emerged. First, sample sizes were generally small, often fewer than 40 participants per group, limiting statistical power and raising concerns about representativeness. Attrition further reduced effective sample sizes, particularly in multi-week protocols, undermining the robustness of findings. Second, most studies relied on homogeneous samples, predominantly female, White, and highly educated, thereby restricting the generalizability of results to more diverse bereaved populations. Third, grief-specific outcome measures were inconsistently employed: fewer than half of the studies used validated grief scales such as the PG-13, ICG, ICG-R, TRIG, or PGS, while others relied exclusively on indirect indicators (e.g., depression, PTSD, mindfulness, or self-compassion). This limits comparability across trials and weakens the possibility to isolate intervention effects on grief per se. Fourth, follow-up assessments were rare and typically short-term, with most studies evaluating outcomes only immediately post-intervention; sustained effects beyond three to six months remain largely undocumented. Finally, comparators were often weak or absent, as only a handful of studies included active control conditions (e.g., grief-focused CBT, PMR), while many adopted uncontrolled pre–post designs.

Taken together, the current evidence base suggests that MBIS for bereavement are promising, but the methodological quality remains limited. Stronger conclusions will require well-powered randomized controlled trials, the systematic use of grief-specific outcome measures, longer follow-up periods, and greater diversity in participant samples. Addressing these limitations is essential to establish the efficacy and clinical applicability of mindfulness-based approaches in bereavement care.

In summary, the 17 publications analyzed in this review provide preliminary but encouraging evidence regarding the feasibility, safety, and potential efficacy of MBIs in bereavement care. MBSR and MBCT emerged as the most established protocols, showing improvements in grief-related distress, depressive symptoms, and overall psychological well-being across heterogeneous loss contexts. Reported therapeutic benefits were generally associated with reductions in maladaptive cognitive processes, such as rumination and experiential avoidance, alongside gains in self-compassion and emotion regulation. Neuroimaging findings, although limited, suggest that symptomatic improvements may be linked to enhanced attentional flexibility and reduced activity in neural regions implicated in affective and self-referential processing, including the posterior cingulate cortex and the thalamus.

Despite these promising findings, the evidence base remains largely composed of pilot and quasi-experimental studies, with only one study meeting high methodological quality criteria. Additionally, several investigations lacked grief-specific outcome measures or long-term follow-up assessments. Therefore, while MBIs show potential as adjunctive interventions, further high-quality randomized controlled trials with diverse samples and standardized grief assessments are needed to clarify their long-term efficacy and clinical positioning alongside established grief therapies.

## 4. Discussion

This systematized narrative review synthesized the emerging evidence on Mindful-ness-Based Interventions (MBIs) in bereavement. The most consistent findings were re-ported for Mindfulness-Based Stress Reduction (MBSR) and Mindfulness-Based Cognitive Therapy (MBCT), which demonstrated preliminary but encouraging effects on grief-related distress, depressive symptoms, and psychological well-being across diverse bereaved populations [40,47]. Despite these promising signals, the evidence base is constrained by several methodological limitations. Many studies relied on small sample sizes (e.g., [26,40]), limiting generalizability and statistical power. In addition, long-term follow-up assessments were often absent, making it difficult to evaluate the durability of treatment effects [51,89]. Another recurring issue is the lack of standardized grief-specific outcome measures, with many studies focusing on general indicators of psychological distress rather than validated grief instruments [26,32], thus reducing comparability across trials.

### 4.1. Theoretical Implications

From a theoretical perspective, MBIs integrate therapeutic elements that appear relevant for both normative grief and PGD [32]. Their efficacy in grief contexts largely derives from enhancing emotion regulation and transforming individuals’ relationship with painful thoughts and feelings [40,53]. Three core mechanisms can be highlighted.

First, acceptance and non-judgmental awareness—defined as intentional present-moment attention with openness and curiosity [51]—counteracts experiential avoidance, a maladaptive coping strategy strongly implicated in complicated grief [32]. For example, In Selah retreats for bereaved parents, the “being with grief” phase explicitly encourages turning toward painful emotions non-judgmentally. Participants reported that this approach helped them “befriend” their grief; for instance, one parent noted that while the grief remained, they “don’t fear it anymore” and stopped trying to “stuff” their tears [51]. The ATTEND model emphasizes attunement and validation of the client’s affective states. By creating a safe environment where “everybody has cried at least once” without judgment, mourners can move away from social and internal “oppression” that forces them to “move on” before they are ready [52]. This orientation increases emotional tolerance and reduces symptom severity, as shown in suicide-bereavement retreats where the non-judging facet of mindfulness improved significantly [49,50]. For example, Panta Rhei retreats resulted in a significant decrease across all measured areas of psychological distress, including tension-anxiety, depression, and anger-hostility [49,50].

Neurobiological evidence increasingly supports the hypothesis that MBIs may alleviate grief-related distress through mechanisms of acceptance and non-judgment. Functional Magnetic Resonance Imaging (fMRI) studies indicate that MBCT enhances executive control and reduces emotional interference in regions implicated in affective and self-referential processing, including the Posterior Cingulate Cortex (PCC) and thalamus [39]. Longitudinal findings further show that MBCT reorganizes resting-state functional connectivity, particularly by reducing internetwork connectivity within the Default Mode Network (DMN) and salience networks [40].

A central mechanism underlying mindful acceptance concerns the distinction between primary affective signals and secondary cognitive reactivity. In line with the classical metaphor of the “two arrows,” the first arrow represents the raw emotional pain (e.g., sudden waves of sadness or longing), whereas the second arrow consists of evaluative judgments and resistance (e.g., “this is unbearable,” “it will never end”) that amplify distress [90]. Emerging neuroscientific evidence suggests that mindfulness reduces this secondary amplification process. Specifically, mindful acceptance appears to down-regulate affective distress via bottom-up mechanisms, modulating primary affective representations without relying predominantly on top-down prefrontal cognitive control [90]. Ap-plied to bereavement, this mechanism suggests that grief-related emotional pain may be recontextualized not as an overwhelming or identity-defining catastrophe, but as a transient and natural affective state. Rather than suppressing grief experiences, mindful acceptance modifies the initial appraisal of affective significance, allowing the experience to unfold without secondary resistance [40,90]. For example, interviewees reported learning that sadness did not need to be fought or suppressed, recognizing instead that “I do not need to battle the sadness, because this is how it must be … if I try to fight it, I do not necessarily feel better” [44].

This process can be conceptually aligned with the Dual Process Model of Coping with Bereavement [20,91], which posits an adaptive oscillation between loss-oriented and restoration-oriented processes. By reducing cognitive elaboration and experiential avoidance, mindfulness may facilitate a more flexible engagement with loss-oriented distress while preventing rigid over-immersion or chronic avoidance. In this sense, mindful acceptance may support the oscillatory regulation described in the Dual Process Model, attenuating prolonged psychophysiological activation and fostering adaptive integration of the loss experience. This oscillatory dynamic is conceptually aligned with the Selah model, which explicitly describes an oscillation between self-focused and other-focused modes of grieving (e.g., being with, surrendering to, and doing with grief). Such oscillatory flexibility prevents rigid entrenchment in distress while also discouraging defensive disengagement from the loss. Notably, participants described that, after “making space” for their pain, they experienced a renewed interest in work and other daily activities, reflecting a fluid transition toward restoration-oriented engagement [51,52].

Second, decentering and attentional regulation allow mourners to observe thoughts and emotions as transient mental events, thereby interrupting grief rumination, another key maintaining factor of PGD [32,42]. Decentering refers to the capacity to experience thoughts and affective states as objective and passing phenomena rather than as accurate reflections of reality or of the self [31,92,93]. This shift reduces cognitive fusion and promotes what has been described as cognitive defusion—viewing catastrophic or absolute thoughts (e.g., “I cannot continue my life without him/her”, “Life has permanently lost its meaning”, etc.) as mental events rather than truths [92,94].

Grief-related rumination is characterized by repetitive, prolonged, and recurrent thinking focused on the loss and its consequences, and represents a key maintaining fac-tor in prolonged grief symptomatology [55]. Mindfulness may interrupt this cycle through attentional training mechanisms [93]. Specifically, mindfulness practice cultivates the capacity to notice when the mind drifts toward ruminative content and to intentionally redirect attention toward a neutral present-moment anchor, such as the breath or bodily sensations [31,95]. This repeated shifting of attentional focus strengthens cognitive flexibility and may reduce automatic immersion in grief-related thought patterns. For example, qualitative evidence from the Existential Behavioral Therapy (EBT) model (which heavily utilizes MBCT exercises) shows that participants use present-moment anchors—such as the breath or nature (the smell of woods, the reflection of trees on water)—to exit the “vicious cycle” of ruminative thinking [44]. Through the development of “letting go,” individuals learn not to suppress intrusive thoughts but to disengage from rigid cognitive loops, thereby weakening maladaptive perseverative processing [93]. This form of psychological distancing may facilitate gradual acceptance of the reality of the loss without reinforcing avoidance [95,96]. Importantly, the notion of “letting go” in mindfulness should not be conflated with the classical psychoanalytic idea of withdrawing libidinal investment from the deceased, as described by Freud [97]. Contemporary bereavement theory has challenged the assumption that adaptive mourning requires detachment. For instance, the Continuing Bonds perspective posits that maintaining an on-going inner relationship with the deceased can represent a protective and integrative process, involving a transformation from love in physical presence to love in symbolic or internalized absence [22]. Within this framework, mindful letting go refers not to relinquishing the bond with the deceased, but to releasing maladaptive cognitive entanglement, thereby allowing the relationship to be integrated in a more flexible and less distressing form.

Neuroimaging findings provide biological support for the proposed role of mindfulness in reducing grief-related rumination and cognitive fusion. For instance, MBCT has been shown to enhance executive control and emotion regulation, with changes in resting-state connectivity linked to improved attentional flexibility [39,40,94]. At the attentional level, mindfulness practice strengthens the anterior cingulate cortex (ACC), a region central to conflict monitoring and attentional regulation [31]. A more efficient ACC may facilitate early detection of attentional drift toward ruminative content, enabling flexible redirection toward present-moment awareness. In this way, neuroplastic changes associated with mindfulness training support decentering and cognitive defusion, weakening the “thought trap” characteristic of grief rumination. The cultivation of meta-awareness creates a psychological “gap” between stimulus and response, allowing painful memories or affective surges to be recognized without immediate immersion [92,94]. In parallel, mindfulness may shift processing from a narrative, self-referential mode toward a more experiential mode of awareness, reducing the subjective and identity-based centrality of grief experiences [31,92,96].

From a constructivist and meaning-centered perspective, the mechanisms described above can be coherently interpreted within Neimeyer’s theory of meaning reconstruction, which conceptualizes bereavement as an active process of rebuilding disrupted assumptive worlds rather than merely attenuating symptoms [21,98]. Grief-related rumination often reflects an attempt to restore coherence in a disrupted assumptive world; however, when rigid and self-referential, it may instead perpetuate distress. Through decentering and attentional flexibility, mindfulness weakens cognitive fusion with catastrophic or identity-defining narratives, thereby creating psychological space for alternative meaning-making processes to emerge. Neurocognitive adaptations may support this shift from perseverative narrative processing toward a more flexible, experiential engagement with loss. In this sense, mindfulness does not eliminate the search for meaning; rather, it trans-forms the mourner’s relationship to meaning-making itself, allowing reconstruction to unfold without the rigid amplification characteristic of prolonged grief. For example, in the study by [52], bereaved parents described a profound shift in self-appraisal: they moved from perceiving themselves as “crazy” for the intensity of their grief to recognizing that “I suffer because I loved” [52]. This reframing represents an instance of meaning reconstruction, whereby emotional pain is no longer interpreted as a pathological symptom requiring elimination, but as a coherent and intelligible response to attachment and love. In this sense, grief becomes recontextualized not as evidence of dysfunction, but as testimony to the enduring relational bond.

Third, self-compassion emerges as a resilience factor, associated with adaptive psychological functioning and reduced self-criticism [94,99]. Interventions embedding compassion practices foster common humanity, mitigating isolation and stigma, and supporting bereaved families in contexts of traumatic loss [99].

Guilt and shame frequently play a central and often debilitating role in bereavement, acting as significant maintaining factors in prolonged grief symptomatology [100]. While guilt typically involves negative evaluation of a specific behavior (e.g., “I should have done more”), shame reflects a global negative evaluation of the self, often accompanied by feelings of worthlessness, powerlessness, and withdrawal. These affective states may obstruct adaptive mourning by predicting more intense grief reactions and elevated symptoms of anxiety, depression, and post-traumatic stress [101]. In cases of suicide bereavement, guilt and shame may be further exacerbated by social stigma and perceived abandonment, leading survivors to conceal the cause of death and to withdraw socially [49,50]. Cultural expectations regarding what constitutes “normal” grieving can additionally intensify shame when individuals feel unable to meet perceived standards of recovery [101]. Within this framework, MBIs cultivate self-compassion through three interrelated components: non-judgmental awareness, self-kindness, and common humanity [81]. Mindful awareness enables individuals to observe guilt- and shame-related thoughts as transient mental events rather than as objective truths or reflections of a fixed identity, thereby reducing over-identification with a “defective self” [96]. Self-kindness directly counteracts harsh self-criticism by fostering an attitude of care toward one’s own suffering. Instead of engaging in punitive internal dialogue, bereaved individuals are encouraged to relate to themselves with the same compassion they would extend to a close friend in distress [99]. The recognition of common humanity reduces the isolating and stigmatizing effects of shame. Acknowledging that suffering, imperfection, and loss are universal aspects of the human condition can normalize grief reactions and restore a sense of belonging, thereby buffering against social withdrawal [99]. For example, The Panta Rhei retreats explicitly utilize the exercise “How would I treat a friend?” to help participants realize the disparity between the compassion they offer others and the harshness they direct toward themselves [49,50].

Beyond its psychological effects, emerging neuroscientific evidence suggests that self-compassion is associated with measurable neural and psychophysiological adaptations. At the structural level, higher self-compassion has been linked to reduced fractional anisotropy in the right superior longitudinal fasciculus (SLF), a white-matter tract connecting the inferior parietal lobule (IPL) and prefrontal regions [102]. Given that the IPL is a key node of both the mirroring network and the Default Mode Network (DMN), these findings suggest that self-compassion may attenuate maladaptive self-referential processing and mind-wandering, thereby reducing grief-related rumination. The mirroring network—comprising the inferior frontal gyrus (IFG), IPL, insula, and anterior cingulate cortex (ACC)—is classically implicated in empathy toward others [102]; generating compassion toward oneself may recruit similar circuits, enabling bereaved individuals to re-late to their own suffering with care and understanding rather than self-criticism.

At the functional level, prolonged grief has been associated with hyperactivation of DMN regions, particularly the posterior cingulate cortex (PCC) and thalamus, which sustain intrusive self-referential thoughts and affective distress [39,40]. As a core component of mindfulness-based approaches, self-compassion may facilitate a shift from a narrative-evaluative self toward an experiential mode of awareness [31]. Compassion-focused practices have been associated with reduced PCC and thalamic activity, suggesting diminished emotional interference and attenuation of catastrophic self-narratives related to the loss [39].

Self-compassion also appears to modulate the autonomic threat system. It has been proposed as a stronger predictor of vagally mediated heart rate variability (vmHRV) than mindfulness alone, indicating enhanced psychophysiological flexibility and stress regulation [103]. Neuroimaging studies further suggest that adopting a compassionate stance toward one’s own suffering down-regulates neural markers of negative affect, whereas self-criticism and self-blame activate threat-related regions such as the amygdala and dorsal ACC [102,103]. In bereavement contexts characterized by intense guilt or shame, this regulatory shift may buffer against sustained threat activation.

Finally, MBIs often integrate holistic mind–body practices, such as yoga or other MBSR exercises, which reduce stress and depressive symptoms in perinatal bereavement and other traumatic losses [26,41,43,48]. Improvements in sleep quality, frequently disrupted in grief, may further mediate these benefits [26,39,54].

In sum, MBIs provide tools to address maladaptive grief processes—particularly avoidance and rumination [32,104]—while enhancing acceptance, decentering, and self-compassion. By doing so, they may not only alleviate suffering but also foster post-traumatic growth and meaning reconstruction in bereavement [105].

### 4.2. Clinical Implications

Mindfulness-based approaches may be especially appropriate in cases of traumatic or high-risk bereavement. Emerging evidence suggests potential benefits for mothers experiencing stillbirth or early pregnancy loss [37,46,48], as well as for individuals be-reaved by suicide, where shame, stigma, and self-blame are particularly prominent [49,50]. In such contexts, mindfulness and self-compassion practices may help regulate intense affective arousal, reduce maladaptive cognitive loops, and support gradual emotional integration.

These interventions may also serve as a secondary preventive function for individuals presenting with elevated grief-related distress who do not yet meet full diagnostic criteria for PGD. Early incorporation of mindfulness-based strategies may reduce rumination, experiential avoidance, and emotional dysregulation, potentially mitigating progression toward more persistent symptomatology [42].

Mindfulness-based approaches may further be considered when grief-focused cognitive-behavioral therapy (CBT) becomes clinically stalled due to the overwhelming intensity of trauma-related affect. In such cases, mindfulness does not require immediate explicit confrontation with loss-related material but instead cultivates distress tolerance and pre-sent-moment awareness, which may enhance emotional regulation and prepare patients for subsequent trauma-focused work [38].

Nevertheless, clinical timing remains crucial. In the very acute phase of bereavement (generally within the first three months), intensive open-monitoring or deep experiential practices may be less appropriate than interventions emphasizing emotional containment, stabilization, and relational support [50]. Gradual, titrated introduction of brief and structured mindfulness exercises may therefore be preferable, ensuring that engagement with painful affect remains within tolerable limits.

The clinical implementation of mindfulness-based approaches in bereavement care requires both therapist preparation and the use of structured intervention models. A foundational element is the therapist’s own contemplative practice. Regular personal engagement in mindfulness meditation may enhance attunement, empathic presence, and affect regulation within the therapeutic relationship, thereby modeling embodied awareness and emotional containment for the bereaved individual [38]. Such therapist presence is particularly relevant in grief work, where relational safety and emotional resonance are central.

Structured frameworks may guide clinical applications. The ATTEND model provides a relationally grounded, mindfulness-informed approach to traumatic bereavement [53,106]. This model emphasizes compassionate presence, validation, and psychoeducation about grief processes, integrating mindfulness principles into a coherent therapeutic stance.

Specific practices can be flexibly incorporated depending on clinical presentation:

- The 3-Minute Breathing Space may be used to help patients regulate acute waves of emotional activation, fostering brief but structured moments of grounding and attentional stabilization [89,105].
- Loving-Kindness (Metta) meditation may support the transformation of anger, resentment, or self-directed hostility, particularly in contexts of traumatic or stigmatized loss [49,50].
- Self-compassion–focused exercises (e.g., structured cognitive reframing practices aimed at identifying self-critical “thought traps”) may be especially helpful for be-reaved clients struggling with guilt and self-blame [99].

In addition, integrating mind–body techniques may enhance accessibility for patients who initially find seated meditation difficult or destabilizing. Progressive Muscle Relaxation (PMR), for example, has demonstrated benefits in bereaved populations and may serve as a somatic entry point to emotion regulation by reducing physiological tension associated with grief [42]. Such integration underscores the importance of tailoring interventions to the individual’s regulatory capacity and readiness.

Overall, mindfulness-based grief interventions appear most effective when delivered within a relationally attuned, structured, and gradually introduced clinical framework rather than as decontextualized meditation practices.

### 4.3. Future Directions

These limitations highlight important directions for future research. Larger, methodologically rigorous randomized controlled trials (RCTs) are needed to establish the efficacy of MBIs for bereavement, particularly in populations at risk for PGD [106]. Future research should include direct comparisons with established “gold standard” treatments, such as grief-focused cognitive behavioral therapy (GF-CBT), to clarify differential efficacy and identify which subgroups may benefit most from each modality. Longitudinal designs with follow-up assessments extending to 6–12 months or beyond are essential to evaluate the durability of treatment gains [24,89].

Second, greater diversification of study populations is warranted. Existing research has relied predominantly on White, female, and highly educated samples, limiting generalizability. Future trials should prioritize the inclusion of males and individuals from di-verse ethnic, racial, and socioeconomic backgrounds. Tailored investigations for high-risk contexts—such as suicide bereavement, gun violence, and perinatal loss—remain particularly important.

Third, refinement of outcome measurement is needed. Researchers should consistently employ validated grief-specific instruments (e.g., PG-13, ICG, TRIG) [32], while integrating multi-method assessments, including physiological markers (e.g., heart rate variability), neuroimaging, and behavioral indices, to complement self-report measures. Beyond symptom reduction, future studies should also assess positive adaptation outcomes, such as life satisfaction, personal growth, and meaning reconstruction.

Fourth, mechanistic and process-oriented research is critical to identify the active ingredients of MBIs. Studies should examine whether specific mindfulness facets (e.g., non-judging, decentering) mediate therapeutic change [26,94], explore dose–response relationships, and clarify the optimal timing of intervention across acute versus chronic phases of bereavement.

Finally, future research should expand to examine systemic and intergenerational impacts. For example, investigating the psychological well-being of partners and children when a family member engages in an MBI may clarify broader family-level effects. In peri-natal loss, longitudinal studies examining outcomes in subsequent pregnancies may pro-vide valuable insight into the enduring protective effects of mind–body interventions.

## 5. Conclusions

Although the current evidence remains limited and heterogeneous, MBIs show promise as adjunctive interventions in bereavement care. By targeting transdiagnostic mechanisms of adaptation, they hold potential to complement established grief therapies and to address unmet needs in populations vulnerable to prolonged or complicated grief. Future high-quality studies are essential to establish their efficacy, clarify their mechanisms of action, and determine their place within evidence-based bereavement interventions.

## Figures and Tables

**Table 1:** Summary of study characteristics.

Author (Year)	Country	Study Design	Population	Type of Loss	Relationship to Deceased	Time Since Loss	Sample Size (*n*)	Intervention Name	Delivery Mode	Duration and Dose	Comparator	Primary Grief Outcome Measure	Secondary Outcome Measures
[37]	Israel	Observational, retrospective cohort	Women bereaved by stillbirth	Stillbirth	Parent	M = 1.79 months (SD = 1.9)	61	Mind-Body Group Therapy—(MBGT)	Group-based, in-person	8 weekly sessions, 2 h each	None	None (no direct grief measure)	EPDS; STAI; PCL-5
[38]	Australia	Randomized controlled trial (parallel, single-blind)	Adults with Prolonged Grief Disorder (PGD)	Medical fatality (64.0%), suicide (28.0%), and unintentional injury (8.0%)	Parent (52.0%), child (22.0%), partner (14.0%), sibling (10.0%), and grandparent (2%)	M = 46.8 months (SD = 43.4)	100 (MBCT = 50, Grief-focused CBT = 50)	Mindfulness-Based Cognitive Therapy (adapted for grief)	Individual, in-person	11 weekly sessions, 90-min each	Grief-focused Cognitive Behavioral Therapy (active comparator)	PG-13; GCQ	BDI-2; BAI; WHOQOL-BREF
[39]	Taiwan	Within-subject design	Bereaved adults	Information not provided	Significant relative	Within 6 months to 4 years	20	MBCT plus an additional 2-h psychoeducation session on loss	Group-based, in-person	8 weekly sessions, 2.5 h each + extra 2-h psychoeducation	None	TRIG	GAD-7; Taiwan Depression Scale; DERS; FFMQ
[40] *	Taiwan	Within-subject design	Bereaved adults	Information not provided	Significant relative	Within 6 months to 4 years	19	MBCT plus an additional 2-h psychoeducation session on loss	Group-based, in-person	8 weekly sessions, 2.5 h each + extra 2-h psychoeducation	None	TRIG	GAD-7; Taiwan Depression Scale; DERS; FFMQ
[41]	USA	Within-subject design	Bereaved adults	Traumatic (i.e., gun violence)	Immediate family member (unspecified)	Information not provided	24	Mindfulness-Based Stress Reduction (MBSR)	Group-based, online	8 weeks; 8 × 2.5-h weekly sessions + 7-h retreat	None	ICG	TSC-40; PCL-5; BDI-2; Pittsburgh Sleep Quality Index; Satisfaction with Life Scale; FFMQ
[42]	USA	Quasi-randomized controlled trial	Widowed adults	Information not provided	Partner/spouse	M = 14.46 months (SD = 8.82)	95 (MT = 37, PMR = 35, WL = 22)	Mindfulness Training (MT)	Group-based, in-person	6 weekly sessions, 2 h each	Progressive Muscle Relaxation (PMR); Wait-list (WL)	ICG-R; YSL; UGRS	Intervention Acceptability; EQ-D
[43] *	USA	Quasi-randomized controlled trial	Widowed adults	Information not provided	Partner/spouse	M = 14.46 months (SD = 8.82)	95 (MT = 37, PMR = 35, WL = 22)	Mindfulness Training (MT)	Group-based, in-person	6 weekly sessions, 2 h each	Progressive Muscle Relaxation (PMR); Wait-list (WL)	None (no direct grief measure)	CES-D; PANAS-X; MAAS; PACT-flexibility; PSS; PSQI; SWLS; UCLA Loneliness Scale
[44]	Germany	Qualitative study	Adults, bereaved partners of palliative patients	Illness	Partner/spouse	M = 17.4 months (SD = 2.7)	16	Existential Behavioral Therapy (EBT)	Group-based, in-person	6 weekly sessions, 3.40 h each	None	Semi-structured interviews	None
[45]	Germany	Randomized Controlled Trial (RCT)	Adults, bereaved partners of palliative patients	Illness	Partner/spouse	71.6% of participants (N = 130) were bereaved at baseline	93	Existential Behavioral Therapy (EBT)	Group-based, in-person	6 weekly sessions, 3.40 h each	None	None (no direct grief measure)	CAMS-R; BSI; WHOQOL-BREF; SWLS; SMiLE
[46]	Iran	Randomized Controlled Trial (RCT)	Women with early pregnancy loss	Early pregnancy loss (miscarriage)	Parent	Information not provided	106 (MBSR = 53, Routine post-pregnancy care = 53)	Mindfulness-Based Stress Reduction (MBSR)	Group-based, in-person	8 weekly sessions, 2 h each	Routine post-pregnancy care	None (no direct grief measure)	DASS-21
[47]	Denmark	Non-randomized controlled pilot study	Bereaved partners	Information not provided	Partner/spouse	Approximately 4 years post-loss	30 (MBCT = 12, Wait list = 18)	MBCT + 2 booster sessions	Group-based, in-person	8 weekly sessions, 2 h each + 2 Booster Sessions	Wait-list	ICG-R	BDI -2; HTQ; CES; LNSeq
[48]	India	Pre–post observational study	Women bereaved by stillbirth	Stillbirth	Parent	M = 14.2 months	36	MBSR	Group-based, in-person	5 weekly sessions	None	PGS	HSCL-10; SWLS; Brief RCOPE; SPS; FFMQ-SF
[49]	Italy	Prospective longitudinal non-randomized intervention study	Adults bereaved by suicide	Suicide	Partner/Spouse (20; 32.8%); Child (21; 34.4%); Sibling (12; 19.7%); Parent (3; 4.9%); Other close relatives (5; 8.2%).	M = 840 days (range 68–11,285)	61	Mindfulness-based weekend retreat (“Pantha Rei”)	Group-based, in-person	Weekend retreat, total 16 h	None	None (no direct grief measure)	FFMQ; SCS; POMS
[50] *	Italy	Non-randomized controlled intervention trial	Adults bereaved by suicide	Suicide	Son/daughter = 35 (36.1%); Father/mother = 8 (8.2%); Brother/sister = 18 (18.5%); Spouse/partner = 29 (29.9%); Other loved one = 7 (7.2%)	Median: 840 days	147 (Intervention Group = 97; Control Group = 50)	Mindfulness-based weekend retreat (“Pantha Rei”)	Group-based, in-person	Weekend retreat, total 16 h	Passive control group (bereaved parents from online support forum, no intervention)	None (no direct grief measure)	FFMQ; SCS; POMS
[51] *	USA	Quasi-experimental	Bereaved parents	Accident/suicide/homicide = 11 (44%); Illness/anomaly = 7 (28%); Unknown = 7 (28%)	Parent	M = 4.74 years (SD = 5.07)	25 (Experimental group)	Mindfulness-based retreat (Selah model)	Group-based, in-person	4 days	Passive control group (bereaved parents from online support forum, no intervention)	None (no direct grief measure)	IES-R; HSCL-25; FFMQ; SCS-SF
[52] *	USA	Qualitative study	Bereaved parents	Accident/suicide/homicide = 5 (26.3%); Illness/anomaly = 6 (31.6%); Unknown = 8 (42.1%)	Parent	M = 5.03 years (SD = 5.21)	19	Mindfulness-based retreat (Selah model)	Group-based, in-person	4 days	None	Semi-structured interviews	None
[53]	USA	Quasi-experimental	Bereaved parents	Death of child: 81%	Parent	M = 1.71 years (SD = 1.98)	42	Mindfulness-based intervention (ATTEND Model)	Individual therapy, in-person	M = 19.18 week (SD = 10.68)	None	None (no direct grief measure)	IES-R; HSCL-25

Notes: Studies marked with an asterisk (*) refer to publications based on the same participant cohort or subsamples. These reports provide complementary findings (e.g., quantitative and qualitative analyses, or different outcome domains) and were synthesized narratively without double-counting participants. For acronyms of Interventions and outcome measures, please refer to the main text. Abbreviations: BAI, Beck Anxiety Inventory; BDI-II (BDI-2), Beck Depression Inventory-II; BSI, Brief Symptom Inventory; CAMS-R, Cognitive and Affective Mindfulness Scale–Revised; CBT, Cognitive Behavioral Therapy; CES, Centrality of Event Scale; CES-D, Center for Epidemiologic Studies Depression Scale; DASS-21, Depression Anxiety Stress Scales-21; DERS, Difficulties in Emotion Regulation Scale; EBT, Existential Behavioral Therapy; EPDS, Edinburgh Postnatal Depression Scale; EQ-D, Experiences Questionnaire—Decentering subscale; FFMQ, Five Facet Mindfulness Questionnaire; FFMQ-SF, Five Facet Mindfulness Questionnaire—Short Form; GAD-7, Generalized Anxiety Disorder-7; GCQ, Grief Cognitions Questionnaire; HSCL-10/HSCL-25, Hopkins Symptom Checklist (10-/25-item versions); HTQ, Harvard Trauma Questionnaire; ICG, Inventory of Complicated Grief; ICG-R, Inventory of Complicated Grief—Revised; IES-R, Impact of Event Scale–Revised; LNSeq, Letter–Number Sequencing; MAAS, Mindful Attention Awareness Scale; MBCT, Mindfulness-Based Cognitive Therapy; MBGT, Mind–Body Group Therapy; MBSR, Mindfulness-Based Stress Reduction; MT, Mindfulness Training; PANAS-X, Positive and Negative Affect Schedule—Expanded Form; PACT-flexibility, Perceived Ability to Cope with Trauma scale—Flexibility subscale; PCL-5, PTSD Checklist for DSM-5; PG-13, Prolonged Grief Disorder-13; PGD, Prolonged Grief Disorder; PGS, Perinatal Grief Scale; POMS, Profile of Mood States; PMR, Progressive Muscle Relaxation; PSQI, Pittsburgh Sleep Quality Index; PSS, Perceived Stress Scale; RCT, randomized controlled trial; RCOPE (Brief RCOPE), Brief Religious Coping; SCS, Self-Compassion Scale; SCS-SF, Self-Compassion Scale—Short Form; SMiLE, Schedule for Meaning in Life Evaluation; SPS, Social Provisions Scale; STAI, State–Trait Anxiety Inventory; SWLS, Satisfaction With Life Scale; TRIG, Texas Revised Inventory of Grief; TSC-40, Trauma Symptom Checklist-40; UGRS, Utrecht Grief Rumination Scale; WL, wait-list; WHOQOL-BREF, World Health Organization Quality of Life—BREF; YSL, Yearning in Situations of Loss scale.

**Table 2:** Main findings, secondary observations, adverse events, and methodological notes from the included studies.

Author (Year)	Main Findings	Other Findings	Adverse Events	Notes
[37]	Although no grief-specific outcome measures were included, the study conceptualized stillbirth as an emotionally traumatic event compounded by disenfranchised grief, stigma, and relational stress. The mind-body group therapy was associated with significant reductions in depressive symptoms, state anxiety, post-traumatic stress symptoms, and suicidal ideation.	Depressive symptoms significantly decreased (EPDS: M = 14.2 to 10.1, *p* < 0.001, d = 0.89), with rates above cutoff dropping from 60.7% to 23.3%. State anxiety also declined (STAI-S: M = 40.6 to 34.5, *p* < 0.001, d = 0.49), with clinical cases decreasing from 77.5% to 54.1%. Post-traumatic stress symptoms showed a smaller but significant reduction (PCL-5: M = 31.4 to 26.4, *p* < 0.001, d = 0.36), with rates above cutoff falling from 36.1% to 21.3%. Suicidal ideation decreased from 19.7% to 8.2% (*p* = 0.02). No significant change was found for trait anxiety (STAI-T, *p* = 0.61). Greater baseline severity predicted stronger improvement; SSRI use and fewer children were associated with greater gains in depression and PTSS, while time since stillbirth predicted improvement in PTSS only.	None reported	
[38]	Both GF-CBT and MBCT produced significant reductions in PGD symptoms at 6 months (large within-group effect, d = 1.2). However, GF-CBT showed superior efficacy, with greater reductions in PGD severity (mean difference = 7.1; *p* = 0.01; d = 0.8) and grief-related cognitions (mean difference = 14.4; *p* = 0.02; d = 0.7). No between-group differences were observed immediately post-treatment, indicating that the superiority of GF-CBT emerged only at follow-up, likely through stronger effects on maladaptive cognitions.	GF-CBT produced greater reductions in depression at 6 months than MBCT (mean difference = 6.6; *p* = 0.04; d = 0.6), an effect independent of comorbid major depression and possibly linked to CBT-specific components (e.g., goal setting, behavioral activation). No between-group differences emerged for anxiety or quality of life, with both interventions yielding comparable improvements in these domains at follow-up.	No adverse events attributed to the interventions were reported	MBCT adapted specifically for prolonged grief; trial highlights MBCT effectiveness but with lower efficacy compared to specialized grief-focused CBT at follow-up. Sociodemographic and loss-related characteristics reported here refer to participants in the MBCT arm (*n* = 50) of the trial, not the full sample
[39]	TRIG scores decreased significantly following MBCT. Mean scores declined from 49.80 (SD = 13.47) at baseline to 37.95 (SD = 12.58) post-intervention, t(19) = −3.98, *p* < 0.001, with a Cohen’s d of −0.89, indicating a robust effect in alleviating grief symptoms.	Post-intervention mindfulness (T-FFMQ) was negatively correlated with grief severity (TRIG-Present; r = −0.52, *p* < 0.05), suggesting that higher mindfulness was associated with lower grief. Neurophysiological data indicated significant positive associations between TRIG scores and activity in the posterior cingulate cortex (PCC; r = 0.34, *p* < 0.04) and thalamus (r = 0.33, *p* < 0.05) during the numerical Stroop task. These associations diminished following MBCT, consistent with reductions in grief severity.	None reported	Sociodemographic characteristics (age and gender) were reported only for the originally recruited sample of 23 participants. Data specific to the final analytical sample (*n* = 20) who completed MBCT were not provided.
[40] *	Mean TRIG scores decreased from 48.74 pre-MBCT to 36.74 post-MBCT, a statistically significant reduction (t = −3.83, *p* = 0.001), indicating substantial alleviation of grief symptoms following the intervention.	Enhanced connectivity between the caudate and CON/SMN networks was positively associated with mindfulness gains and negatively with anxiety and emotion dysregulation, suggesting improved emotional regulation after MBCT. In contrast, increased SMN–VN connectivity was linked to lower mindfulness and higher anxiety and dysregulation, indicating a maladaptive pattern.	None reported	Huang et al. (2021) confirmed significant TRIG reduction after MBCT but reported neural connectivity correlations only for mindfulness, anxiety, and emotion regulation; the study builds on Ref. [39], which demonstrated PCC activity reduction, thus representing complementary analyses on the same bereaved population.
[41]	At baseline, 79% of participants scored above the clinical cutoff for complicated grief, with higher grief severity compared to non–gun violence bereavement samples. After 8 weeks of MBSR, grief scores decreased by 23%, though this reduction did not remain significant after Bonferroni correction. Analyses of changes across intervention dosage (pre-, mid-, post-) revealed no significant effects. Importantly, increases in dispositional mindfulness (FFMQ) predicted greater reductions in grief, suggesting that enhanced mindful awareness may facilitate a more adaptive relationship with loss.	Most pronounced improvements in trauma, PTSD, depression, and sleep difficulties occurred within the first 5 weeks of MBSR; no significant changes from week 5 to week 8.	None reported	One participant was a direct survivor of gun violence (gunshot injury) rather than bereaved. Limitations for grief measure: 11 of 24 participants opted out of the ICG, resulting in smaller sample sizes (N = 13 pre/post, N = 9 mid). Reduced power likely limited detection of significant change in grief.
[42]	Grief severity (ICG-R): both MT and PMR groups showed significant declines up to 1-month follow-up, while the waitlist did not. PMR, but not MT, declined significantly more than WL, with medium between-group effect (d = 0.47). Estimated grief scores in PMR fell below the cutoff for probable complicated grief at follow-up. Secondary/intervening variables included yearning (YSL) and grief rumination (UGRS). Yearning declined in MT and PMR, and was lower in PMR vs. WL (d = 0.65) and MT vs. WL (d = 0.45). Grief rumination declined across all groups without between-group differences.	Interventions were highly acceptable: 97% of completers would recommend the group, and both MT and PMR rated information as highly applicable. For decentering (EQ-D), contrary to hypotheses, PMR and WL—but not MT—showed significant increases up to 1-month follow-up. PMR outperformed MT (*p* = 0.007) and WL, with medium effect sizes (PMR vs. WL d = 0.66; within-group d = 0.72)	None reported	-
[43] *	This secondary analysis builds on findings from the parent trial. The parent trial found that while both MT and Progressive Muscle Relaxation (PMR) groups showed significant rates of decline in primary grief outcomes (grief severity and yearning), only the PMR group showed a greater rate of decline in grief severity compared to the wait-list control group. So, while MT showed promising results for depression, negative affect, and stress in this secondary analysis, its direct impact on primary grief severity as compared to a control group was not as pronounced as PMR in the initial trial	For Mindfulness Training (MT), participants reported significantly lower depressive symptoms than wait-list at post-intervention (t2: Mdiff = −8.84, *p* = 0.002) and at the 1-month follow-up (t3: Mdiff = −6.72, *p* = 0.023). MT also led to significantly lower negative affect at t2 (Mdiff = −4.65, *p* = 0.005) and t3 (Mdiff = −4.32, *p* = 0.008). Perceived stress was significantly lower in MT compared to wait-list only at t3 (Mdiff = −4.06, *p* = 0.023). No significant MT effects were found for coping flexibility, life satisfaction, loneliness, mindfulness (MAAS), positive affect, or sleep quality	None reported	This article is a secondary analysis of the same data and participants from the parent trial by Ref. [42].
[44]	Semi-structured interviews indicated that participants perceived mindfulness and acceptance as particularly helpful self-regulation strategies in coping with bereavement. Mindfulness practice supported stopping ruminative thinking, living in the present, and allowing emotions to come and go without resistance.	Participants highlighted the importance of social support (sharing emotions, sense of belonging, being understood, exchange of coping strategies, group cohesion, continuity with palliative care) and additional self-regulation strategies (focusing on positive memories, finding new sources of strength, pursuing new goals, self-care). These aspects were perceived as highly beneficial and consistent with EBT principles.	None reported	This article refers to a previous randomized controlled trial (RCT) with 160 relatives, which demonstrated long-term positive effects of Existential Behavioral Therapy (EBT) on quality of life and psychological stress reduction Ref. [45].
[45]	71.6% of the 130 participants were bereaved at baseline. Exploratory analyses in the bereaved subsample (*n* = 93) showed similar correlations as in the total sample: higher mindfulness was associated with lower psychological distress and greater well-being, replicating the overall findings	Higher baseline mindfulness correlated with lower distress (r = −0.51) and greater life satisfaction (r = 0.52) and QoL (r = 0.60), including in bereaved relatives. Dispositional mindfulness predicted improvements in distress, QoL, and meaning in life up to 12 months. EBT led to a significant long-term increase in mindfulness (T1/T4, *p* = 0.02), and intervention effects on depression, QoL, and life satisfaction were partly mediated by mindfulness.	None reported	Only 79.7% of the participants in that RCT were bereaved before or during the intervention.
[46]	Although grief was not directly measured, the MBSR intervention significantly reduced anxiety, depression, and stress in women after early pregnancy loss. These improvements in psychological distress may indirectly support the bereavement process.	After 8 MBSR sessions, the intervention group showed significant reductions compared to controls: anxiety (14.34 → 7.90 vs. 13.79; *p* < 0.0001), depression (15.11 → 7.83 vs. 16.26; *p* < 0.0001), and stress (18.39 → 9.26 vs. 18.13; *p* < 0.0001).	None reported	
[47]	No significant effect on complicated grief (Completers: Hedges’ g = 0.02; ITT: Hedges’ g = 0.05, ns)	Depressive symptoms: Significant reduction in completers at 5-month Follow-Up (g = 0.84; interaction g = 0.88, *p* = 0.02); ITT trend (g = 0.49, *p* = 0.065) with medium effect (g = 0.61). Elevated cases dropped from 50% → 0% vs. stable 29% in WL.	None reported	
[48]	Working memory: Pre–post increase in completers (g = 0.62, *p* = 0.04), difference vs. WL at post (*p* = 0.02), but not maintained at Follow-Up; ITT similar trend (g = 0.50, *p* = 0.044).		None reported	
[49]	PTSS: No significant effects (Completers g = 0.24; ITT g = 0.12).	No adverse events directly related to MBCT were reported. Attrition occurred: 25 participants declined (reasons: not interested, illness, mobility issues), and 6 dropped out (hearing impairment, new illness, mobility problems, or “MBCT not for them”).	None reported	Completers = only participants who completed MBCT (*n* = 12). Intention-to-Treat = all participants who started MBCT (*n* = 18), including dropouts.
[50] *	Psychological distress decreased significantly (HSCL-10, *p* = 0.042). Mindfulness facets improved, especially acting with awareness (*p* = 0.033) and describing (*p* = 0.043), though non-reacting declined (*p* = 0.025).	High adherence (~30 min/day), low dropout (10.3%), and strong acceptability were reported.	None reported	Results are based on 26 women (89.7% of those who started) who completed the five-week intervention and post-test, and 23 women (88.5%) who completed the six-week follow-up assessment.
[51] *	Mindfulness- and self-compassion-based retreats led to significant reductions in psychological distress (POMS subscales: tension, depression, anger, fatigue, confusion), decreased over-identification on the SCS, and improved mindfulness (FFMQ “describing”). Multiple retreats yielded further gains in self-kindness and non-judging. Group context promoted acceptance, emotional regulation, and reduced rumination, supporting survivors’ needs for connection and understanding.	None reported	It was observed that sharing sessions and grief-focused practices could “in some cases evoke strong emotions.” This was not reported as an adverse effect but rather as part of a cathartic process occurring in a “safe environment, under the guidance of group leaders with specific expertise” in mindfulness, self-compassion, bereavement, and suicide prevention. Participants were divided into two subgroups: single participation (*n* = 47), those who attended only one weekend retreat, and multiple participation (*n* = 14), those who attended two or more retreats.	Mindfulness- and self-compassion-based retreats led to significant reductions in psychological distress (POMS subscales: tension, depression, anger, fatigue, confusion), decreased over-identification on the SCS, and improved mindfulness (FFMQ “describing”). Multiple retreats yielded further gains in self-kindness and non-judging. Group context promoted acceptance, emotional regulation, and reduced rumination, supporting survivors’ needs for connection and understanding.
[52] *	Significant reduction in psychological distress (POMS: ↓ Tension-Anxiety, Depression, Anger, Fatigue, Confusion; ↑ Vigor; η^2^ = 0.05–0.10) and increases in mindfulness (FFMQ: Observe, Describe, Non-Judge, Non-React; η^2^ = 0.04–0.07) and self-compassion (↑ Self-Kindness, ↓ Overidentification; η^2^ = 0.03–0.05). Broad applicability (no baseline predictors). Intervention tailored to grief pain, shame, guilt, forgiveness; group setting addressed disenfranchised grief. Limitations: non-randomized design, passive control, sample skewed to highly educated females, help-seeking bias.	Newly bereaved individuals by suicide may not benefit from awareness and visualization practices, which could instead overactivate the affective experience (with the risk of backdraft), given their more urgent need for containment and connection.	Five participants described emotional discomfort in response to other participants. Five expressed discomfort with certain retreat activities, such as paired exercises. Two expressed discomfort with the amount of personal disclosure they thought was expected of them. Five described physical discomfort, often in response to sitting for long periods of time. Three experienced some discomfort or distress related to exposure to stories of how other parents’ children had died.	Significant reduction in psychological distress (POMS: ↓ Tension-Anxiety, Depression, Anger, Fatigue, Confusion; ↑ Vigor; η^2^ = 0.05–0.10) and increases in mindfulness (FFMQ: Observe, Describe, Non-Judge, Non-React; η^2^ = 0.04–0.07) and self-compassion (↑ Self-Kindness, ↓ Overidentification; η^2^ = 0.03–0.05). Broad applicability (no baseline predictors). Intervention tailored to grief pain, shame, guilt, forgiveness; group setting addressed disenfranchised grief. Limitations: non-randomized design, passive control, sample skewed to highly educated females, help-seeking bias.
[53]	The intervention group showed significant reductions in trauma, depression, and anxiety at post-test, partly maintained at follow-up (trauma but not depression). Increases in mindfulness (Describe, Act with Awareness) and self-compassion were observed post-test, with a delayed gain in Nonjudging at follow-up.	Sample characteristics refer only to the experimental group that received the mindfulness intervention. Indirect measures of grief-related distress were used, assessing psychological suffering (trauma, anxiety, depression) and well-being (mindfulness, self-compassion) among bereaved parents. These outcomes were considered indicators of grief-related distress.	A number of participant scores slightly increased, indicating intensifying symptoms from pretest to post-test. 14 sets increased: six for the Impact of Event Scale-Revised (IES-R) and eight for the 25-item Hopkins Symptom Checklist (HSCL-25).	The intervention group showed significant reductions in trauma, depression, and anxiety at post-test, partly maintained at follow-up (trauma but not depression). Increases in mindfulness (Describe, Act with Awareness) and self-compassion were observed post-test, with a delayed gain in Nonjudging at follow-up.

Notes: Studies marked with an asterisk (*) are secondary publications or complementary analyses derived from the same participant cohort. They report additional or stratified findings (e.g., quantitative vs. qualitative results, secondary outcomes, or subgroup analyses) and were synthesized narratively without double-counting participants. Abbreviations: BDI-II (BDI-2), Beck Depression Inventory-II; CAMS-R, Cognitive and Affective Mindfulness Scale–Revised; DASS-21, Depression Anxiety Stress Scales-21; EPDS, Edinburgh Postnatal Depression Scale; EQ-D, Experiences Questionnaire—Decentering subscale; FFMQ, Five Facet Mindfulness Questionnaire; GCQ, Grief Cognitions Questionnaire; HSCL-10/HSCL-25, Hopkins Symptom Checklist (10-/25-item versions); HTQ, Harvard Trauma Questionnaire; ICG, Inventory of Complicated Grief; ICG-R, Inventory of Complicated Grief—Revised; IES-R, Impact of Event Scale–Revised; ITT, intention-to-treat; MBCT, Mindfulness-Based Cognitive Therapy; MBSR, Mindfulness-Based Stress Reduction; MT, Mindfulness Training; PCL-5, PTSD Checklist for DSM-5; PG-13, Prolonged Grief Disorder-13; PGS, Perinatal Grief Scale; PMR, Progressive Muscle Relaxation; PSQI, Pittsburgh Sleep Quality Index; PTSS, post-traumatic stress symptoms; RCT, randomized controlled trial; STAI-S/STAI-T, State–Trait Anxiety Inventory (State/Trait forms); SWLS, Satisfaction With Life Scale; TRIG, Texas Revised Inventory of Grief; TSC-40, Trauma Symptom Checklist-40; UGRS, Utrecht Grief Rumination Scale; WL, wait-list; YSL, Yearning in Situations of Loss scale; ns, not significant.

**Table 3:** Summary of effect directions and methodological quality ratings of the included studies.

Study (Author, Year)	Effect Direction (↑ ↓ ↔)	Quality Rating (Green/Yellow)	Key Limitations
[37]	↓ (significant reductions in depression, state anxiety, PTSD symptoms, and suicidality; no change in trait anxiety)	🟡 Yellow (promising improvements with validated tools, but retrospective design, no control group, short follow-up, and no grief-specific outcomes)	Retrospective cohort without control, no grief-specific measure, short-term follow-up
[38]	↓ (both groups improved, GF-CBT > MBCT at 6-month follow-up)	🟢 Green (well-designed RCT, good fidelity, adequate sample size)	High female predominance (87%), only 60% retained at 6-month follow-up, no no-treatment control, therapists not blinded.
[39]	↓ (significant reductions in grief, depression, anxiety, emotion dysregulation; ↑ mindfulness, improved Stroop task performance and fMRI executive control)	🟡 Yellow (good internal validity but no control group, small sample, high female predominance)	No comparator arm, small sample size, self-selection bias, short-term follow-up only, limited generalizability.
[40]	↓ (significant reductions in grief, anxiety, depression, emotion dysregulation; ↑ mindfulness and emotion regulation; fMRI showed decreased default mode and salience connectivity, improved cortico-subcortical regulation)	🟡 Yellow (rigorous intervention and multimodal assessment, but no control group and small sample size)	No comparator arm, small and predominantly female sample, lack of long-term follow-up, high interindividual variability in neural outcomes.
[41]	↓ (significant reductions in trauma, PTSD, depression, sleep difficulties; ↑ life satisfaction, mindfulness; grief reduction trend but not significant after correction)	🟡 Yellow (robust within-subject improvements but uncontrolled design, small sample, high dropout for grief scale, non-representative sample)	No control group, self-report only, grief data incomplete (many opted out), predominantly White and female participants, limited generalizability
[42]	↓ (both MT and PMR reduced grief severity, yearning, and rumination; only PMR > waitlist for grief severity; decentering ↑ in PMR and WL but not in MT)	🟡 Yellow (good design and fidelity, but quasi-randomization, homogeneous sample, and limited follow-up reduce generalizability)	Quasi-random assignment to wait-list, short 1-month follow-up, mostly White older women, reliance on self-report, no monitoring of adverse effects
[43]	↓ (both MT and PMR ↓ depression and negative affect vs. WL; MT ↓ stress at follow-up; no effects on positive affect, mindfulness, coping flexibility, sleep, life satisfaction, or loneliness)	🟡 Yellow (methodologically robust secondary RCT analysis, but downgraded due to absence of grief-specific outcomes in this paper, modest and selective effects, homogeneous sample)	No grief-specific outcomes, small and homogeneous sample (older White widows), attrition and missing data requiring imputation, short follow-up, limited generalizability.
[44]	↑ (participants reported mindfulness and acceptance helped reduce rumination, fostered self-regulation, social support was valued)	🟡 Yellow (rigorous qualitative design and analysis, but small, homogeneous sample; interpretive bias possible)	Limited generalizability, researcher bias risk (familiarity with intervention), qualitative data only, no control group.
[45]	↑ (significant improvements in distress, QoL, meaning in life; mindfulness partly mediated long-term effects)	🟡 Yellow (methodologically robust RCT, but downgraded because no grief-specific outcomes were assessed, limiting direct relevance for bereavement)	No grief-specific outcome measure, heterogeneous sample (caregivers + bereaved)
[46]	↓ (significant reductions in depression, anxiety, and stress in the intervention vs. control group, large effect sizes)	🟡 Yellow (rigorous RCT with significant results, but downgraded due to absence of grief-specific outcome; short follow-up)	No grief measure, only perinatal loss population
[47]	↓ (significant reduction in depressive symptoms in MBCT vs. WL at 5-month FU, g = 0.84–0.88; no significant differences for CG or PTSS; trend toward ↑ working memory)	🟡 Yellow (well-conducted pilot with validated grief measure included, but small sample, high attrition, non-random allocation, limited power)	Small sample, non-random assignment (urban vs. rural), high attrition in intervention, limited generalizability, effects significant only for depression but not grief/PTSS, follow-up effects delayed.
[48]	↓ (significant reductions in perinatal grief and subscales, psychological symptoms; ↑ life satisfaction, some mindfulness facets improved, non-react facet worsened)	🟡 Yellow (promising results and culturally sensitive design, but uncontrolled pilot, small sample, limited generalizability)	No control group, small sample, purposive/snowball sampling, short follow-up, measurement challenges with mindfulness scales due to translation issues.
[49]	↓ (significant reductions in tension, depression, anger, fatigue, confusion; no change in vigor; ↓ overidentification in SCS, slight ↑ in mindfulness facets)	🟡 Yellow (methodologically limited pilot; no grief-specific outcomes, no control group, self-selected participants)	No grief-specific outcome measure, uncontrolled design, small and self-referred sample, short-term assessment only, uncertain generalizability.
[50]	↓ (intervention group showed significant reductions in all POMS distress subscales, ↑ vigor; ↑ mindfulness facets: observe, describe, nonjudge, nonreact; ↑ self-compassion: self-kindness, common humanity, ↓ overidentification)	🟡 Yellow (controlled design with significant improvements, but downgraded due to lack of grief-specific outcome and non-randomized allocation)	No grief-specific outcome measure, non-randomized allocation, passive control group, predominantly female/educated sample
[51]	↓ (significant reductions in trauma, depression, and anxiety in intervention vs. comparison; some transient gains in mindfulness facets and self-compassion; only “nonjudge” facet ↑ significantly at follow-up)	🟡 Yellow (valuable quasi-experimental evidence, but downgraded due to absence of grief-specific outcome measures; high attrition, small intervention sample, non-random allocation, short follow-up)	No grief-specific outcome, non-randomized allocation, short follow-up, high attrition, limited generalizability
[52]	↑ (participants reported greater self-compassion, improved emotional regulation, validation of grief, enhanced continuing bonds, and supportive community; some noted distress from exposure to others’ traumatic stories)	🟡 Yellow (rigorous qualitative methods with reflexivity and triangulation, but small, self-selected, predominantly White female sample; possible researcher bias due to familiarity with participants)	Qualitative design limits generalizability, no standardized grief outcome measures, potential bias from researcher involvement, adverse effects noted for a few participants exposed to others’ traumatic loss stories.
[53]	↓ (significant reductions in PTSD intrusion, avoidance, hyperarousal, and HSCL depression/anxiety; medium-to-large effect sizes, d = 0.70–0.92)	🟡 Yellow (clinically meaningful improvements with validated measures, but downgraded due to no grief-specific outcome, uncontrolled design, retrospective data)	No grief-specific outcomes, uncontrolled chart review design

Notes: Quality rating was assigned using a pragmatic traffic-light system: 🟢 Green = high quality (well-conducted RCT, valid measures, adequate sample size); 🟡 Yellow = moderate quality (methodological limitations such as absence of follow-up, small sample size, or weak comparator. Abbreviations: GF, grief-focused; GF-CBT, grief-focused cognitive behavioral therapy; MBCT, Mindfulness-Based Cognitive Therapy; MBSR, Mindfulness-Based Stress Reduction; MT, Mindfulness Training; PMR, Progressive Muscle Relaxation; PTSD, post-traumatic stress disorder; PTSS, post-traumatic stress symptoms; RCT, randomized controlled trial; WL, wait-list. Symbols: Arrows indicate direction of effect (↑ improvement; ↓ deterioration; ↔ no significant change).

## Data Availability

No new data were created or analyzed in this study. Data sharing is not applicable to this article.

## Abbreviations

The following abbreviations are used in this manuscript:

MBI	Mindfulness-Based Intervention
MBCT	Mindfulness-Based Cognitive Therapy
MBSR	Mindfulness-Based Stress Reduction
ACT	Acceptance and Commitment Therapy
EBT	Existential Behavioral Therapy
PGD	Prolonged Grief Disorder
PG-13	Prolonged Grief Disorder scale (13-item version)
ICG	Inventory of Complicated Grief
TRIG	Texas Revised Inventory of Grief
PGS	Perinatal Grief Scale
FFMQ	Five Facet Mindfulness Questionnaire
SCS	Self-Compassion Scale

## Appendix A

The traffic-light system was used as a pragmatic and integrative approach to summarize the methodological characteristics of heterogeneous studies included in this systematized narrative review. Ratings were assigned based on predefined criteria reflecting study design, sample characteristics, outcome relevance, and follow-up.

**Table A1:** Operational criteria for assigning traffic-light quality ratings to included studies.

Criterion	🟢 Green (Higher-Quality Evidence)	🟡 Yellow (Moderate-Quality Evidence)	🔴 Red (Low-Quality Evidence)
Study design	Randomized controlled trials (RCTs) or well-designed controlled studies with an appropriate comparison group.	Pilot trials, quasi-experimental designs, non-randomized controlled studies, or structured qualitative studies.	Uncontrolled studies, simple pre–post designs, case series, case reports, or purely descriptive studies.
Sample characteristics	Adequate sample size for the study design; clearly defined inclusion and exclusion criteria.	Small or convenience samples; partially described inclusion criteria.	Very small samples or insufficiently described participant characteristics.
Outcome measures	Use of validated, grief-specific outcome measures as primary endpoints.	Outcomes not specifically focused on grief (e.g., general distress, depression, wellbeing) or use of secondary/proxy measures.	Absence of standardized or validated outcome measures; vague or poorly operationalized outcomes.
Follow-up	At least one follow-up assessment beyond immediate post-intervention.	No follow-up or very short follow-up duration.	No follow-up assessment.
Reporting quality	Clear, coherent, and sufficiently detailed reporting of methods and results.	Generally adequate reporting with identifiable methodological limitations.	Insufficient methodological detail or major reporting deficiencies.

Note. The traffic-light classification reflects an integrative appraisal of methodological characteristics and does not constitute a formal risk-of-bias assessment.

## Appendix B

**Table A2:** Full-text articles excluded after eligibility assessment and reasons for exclusion.

Authors (Year)	Title (Shortened)	Journal	Reason for Exclusion
[105]	ATTEND: toward a mindfulness-based bereavement care model	Death studies	Conceptual framework only (ATTEND model), no empirical data.
[54]	Relationship Between Mindfulness and Posttraumatic Stress in Women Who Experienced Stillbirth	Journal of obstetric, gynecologic, and neonatal nursing	Baseline cross-sectional data only, no intervention effects.
[107]	Cognitive behavioural therapy and mindfulness for relatives of missing persons: a pilot study	Pilot and feasibility studies	Population: relatives of long-term missing persons, not bereaved by death.
[108]	Impact of a contemplative end-of-life training program: being with dying	Palliative & supportive care	Population: healthcare professionals, not bereaved individuals.
[109]	Perspectives of bereaved partners of lung cancer patients on the role of mindfulness in dying and grieving: A qualitative study	Palliative medicine	Pre-loss intervention (MBSR before patient’s death), not post-bereavement.
[110]	Mindfulness: existential, loss, and grief factors in women with breast cancer	Journal of psychosocial oncology	Population: women with breast cancer; grief related to illness, not death bereavement.
[111]	Practitioner perspectives on the use of acceptance and commitment therapy for bereavement support: a qualitative study	BMC palliative care	Population: professionals providing bereavement support, not bereaved individuals.
[99]	Self-Compassion for Caregivers of Children in Parentally Bereaved Families: A Theoretical Model and Intervention Example	Clinical child and family psychology review	Theoretical article using Resilient Parenting for Bereaved Families as example, no empirical bereavement data.
[112]	Mindfulness and grief: The MADED program mindfulness for the acceptance of pain and emotions in grief	Psicooncología	Intervention protocol (Mindulfness para la Aceptación del Dolor y las Emociones en el Duelo), no results reported.
[113]	Healing grief—an online self-help intervention programme for bereaved Chinese with prolonged grief: study protocol for a randomized controlled trial	European Journal of Psychotraumatology	Intervention not mindfulness-based (Healing Grief program, mindfulness only mentioned superficially).
[114]	The Effectiveness of Mindfulness Training on the Grieving Process and Emotional Well-Being of Chronic Pain Patients	Journal of Clinical Psychology in Medical Settings	Population not bereaved by death (chronic pain patients, functional/social losses).

ATTEND, Attunement–Trust–Therapeutic touch–Egalitarianism–Nuance–Death education model; MADED, Mindfulness para la Aceptación del Dolor y las Emociones en el Duelo; MBSR, Mindfulness-Based Stress Reduction.
